# Co‐morbidities and co‐medications as confounders of cardioprotection—Does it matter in the clinical setting?

**DOI:** 10.1111/bph.14839

**Published:** 2020-01-03

**Authors:** Petra Kleinbongard, Hans Erik Bøtker, Michel Ovize, Derek J. Hausenloy, Gerd Heusch

**Affiliations:** ^1^ Institute for Pathophysiology, West German Heart and Vascular Center University of Essen Medical School Essen Germany; ^2^ Department of Cardiology Aarhus University Hospital Skejby Aarhus Denmark; ^3^ INSERM U1060, CarMeN Laboratory, Université de Lyon and Explorations Fonctionnelles Cardiovasculaires, Hôpital Louis Pradel, Hospices Civils de Lyon Lyon France; ^4^ Cardiovascular and Metabolic Disorders Program Duke‐National University of Singapore Medical School Singapore; ^5^ National Heart Research Institute Singapore National Heart Centre Singapore; ^6^ Yong Loo Lin School of Medicine National University Singapore Singapore; ^7^ The Hatter Cardiovascular Institute University College London London UK; ^8^ Research and Development The National Institute of Health Research University College London Hospitals Biomedical Research Centre London UK; ^9^ Tecnologico de Monterrey Centro de Biotecnologia‐FEMSA Monterrey Nuevo Leon Mexico

## Abstract

The translation of cardioprotection from robust experimental evidence to beneficial clinical outcome for patients suffering acute myocardial infarction or undergoing cardiovascular surgery has been largely disappointing. The present review attempts to critically analyse the evidence for confounders of cardioprotection in patients with acute myocardial infarction and in patients undergoing cardiovascular surgery. One reason that has been proposed to be responsible for such lack of translation is the confounding of cardioprotection by co‐morbidities and co‐medications. Whereas there is solid experimental evidence for such confounding of cardioprotection by single co‐morbidities and co‐medications, the clinical evidence from retrospective analyses of the limited number of clinical data is less robust. The best evidence for interference of co‐medications is that for platelet inhibitors to recruit cardioprotection per se and thus limit the potential for further protection from myocardial infarction and for propofol anaesthesia to negate the protection from remote ischaemic conditioning in cardiovascular surgery.

**LINKED ARTICLES:**

This article is part of a themed issue on Risk factors, comorbidities, and comedications in cardioprotection. To view the other articles in this section visit http://onlinelibrary.wiley.com/doi/10.1111/bph.v177.23/issuetoc

**Abbreviations**
ACE: angiotensin converting enzyme
ARB: angiotensin receptor blocker
CABG: coronary artery bypass graft
DPP‐4: dipeptidyl peptidase‐4
GIK: glucose–insulin–potassium
GLP‐1: glucagon‐like peptide‐1
LV: left ventricle
LVEF: left ventricular ejection fraction
O‐GlcNAc: O‐linked b‐*N*‐acetylglucosamine
PCI: percutaneous coronary intervention
RIC: remote ischaemic conditioning
SGLT‐2: sodium‐dependent glucose transporter‐2
SPECT: single‐photon emission CT
STAT5: signal transducer and activator of transcription 5
STEMI: ST‐segment elevation myocardial infarction
TIMI: thrombolysis in myocardial infarction

## 1 INTRODUCTION

**Figure 1:**
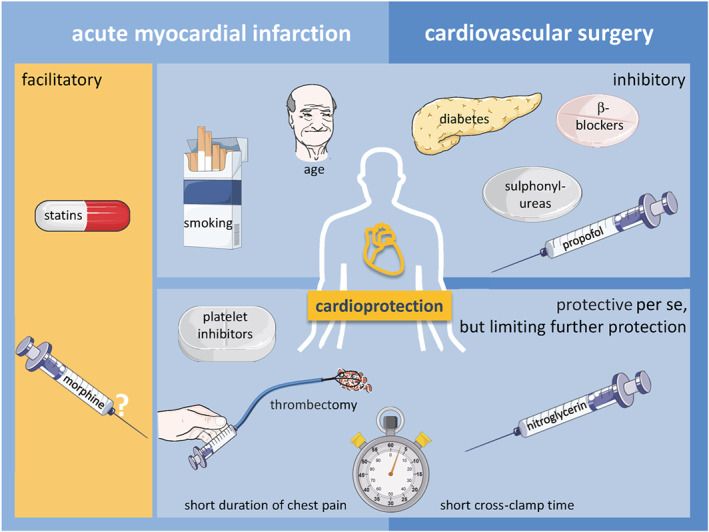
Interference by risk factors, co‐morbidities, and co‐medications with cardioprotection by ischaemic conditioning in patients suffering acute myocardial infarction or undergoing cardiovascular surgery. There may be several scenarios: Cardioprotection can be facilitated (orange background) or inhibited (blue background). Short periods of myocardial ischaemia (i.e., rapid reperfusion) or co‐medications can reduce myocardial damage per se (blue background) and thus limit the potential for further cardioprotection

There is firm evidence that infarct size, as the gold standard endpoint of cardioprotection (Bøtker et al., 2018; Lindsey et al., 2018), can be reduced by cardioprotective interventions such as the ischaemic conditioning phenomena (Hausenloy et al., 2016) and many compounds and drugs (Davidson et al., 2019; Hausenloy et al., 2017; Heusch & Gersh, 2017) which often relate to the signalling steps underlying the conditioning phenomena (Heusch, 2015). Despite all experimental evidence, the translation of cardioprotection to patients and their clinical outcome has been largely disappointing so far (Hausenloy et al., 2013; Hausenloy et al., 2017; Heusch & Rassaf, 2016; Lecour et al., 2014), for a number of conceptual and methodological reasons (Heusch, 2017). Prominent among the reasons which have been proposed to explain the poor translation from cardioprotection by ischaemic and pharmacological conditioning in the experimental setting to patient benefit has been the use of animals (species issue), often of young age (age issue, Boengler, Schulz, & Heusch, 2009) and the lack of typical co‐morbidities (diabetes, hypertension and hyperlipidaemia) and co‐medications (statins, β‐blockers, ACE inhibitors, angiotensin AT_1_ receptor antagonists (ARBs), calcium antagonists, and nitrates) which are characteristic of patients with ischaemic heart disease (Ferdinandy, Hausenloy, Heusch, Baxter, & Schulz, 2014). Indeed, a number of experimental studies have provided evidence that notably diabetes (Whittington, Babu, Mocanu, Yellon, & Hausenloy, 2012), but also hypertension with ventricular hypertrophy (Pagliaro & Penna, 2017), hyperlipidaemia (Adelborg et al., 2017) and also drugs such as statins (Ferdinandy et al., 2014; Schulz, 2005) interfere with cardioprotective signalling. The underlying mechanisms and influences of co‐morbidities and co‐medications on cardioprotective signalling have been reviewed by Andreadou et al. (2017) and Ferdinandy et al. (2014). The interference of co‐morbidities and co‐medications with cardioprotection can occur in different scenarios (Figure 1): (a) co‐morbidities (e.g., diabetes, Wider et al., 2018) and co‐medications (e.g., sulphonylureas, Kottenberg,Thielmann, et al., 2014) can inhibit ischaemic conditioning (Kottenberg, Thielmann, et al., 2014), (b) co‐morbidities (e.g.. pre‐infarction angina, Heusch, 2001) and co‐medications (volatile anaesthesia, P2Y_12_ receptor antagonists s) can induce cardioprotection and then either permit additional protection by ischaemic conditioning (e.g., isoflurane, Kottenberg et al., 2012, or not, Cohen & Downey, 2017), and (c) co‐medications can facilitate cardioprotection by ischaemic conditioning (e.g., statins, Sloth et al., 2015).

While experimental animal studies have provided solid evidence for single confounders of cardioprotection, such “confounded cardioprotection” may again not be readily translatable to clinical practice (Kleinbongard et al., 2016; Sloth et al., 2015). The present review therefore attempts to critically analyse the evidence for confounders of cardioprotection in patients with acute myocardial infarction and in patients undergoing cardiovascular surgery (Table 1).

**Table 1:** Confounders of cardioprotection by ischaemic conditioning in patients with acute myocardial infarction and in patients undergoing cardiovascular surgery

a literature overview	Confounder		Cardioprotection by	Number of patients	Reference
Acute myocardial infarction					
Risk factors/co‐morbidities					
Retrospective	Age (<70/>70 years)	—	RIC	48/23	Sloth et al., 2015
Meta‐analysis	Age (>62 years)	↓	PoCo	560 (total)	Zhou et al., 2012
Retrospective	Age (>65 years)	↓	PoCo	80/35	Darling et al., 2007
Retrospective	Sex (male/female)	—	RIC	57/14	Sloth et al., 2015
Retrospective	Smoking (yes/no)	↓^a^	RIC	34/37	Sloth et al., 2015
Retrospective	Obesity/body mass index (<25/≥25 kg·m^−2^)	—	RIC	27/44	Sloth et al., 2015
Retrospective	Hyperlipidaemia (yes/no)	—	RIC	30/29	Sloth et al., 2015
Retrospective	Hypertension (yes/no)	—	RIC	32/39	Sloth et al., 2015
Pre‐specified	Diabetes (yes/no)	—	RIC	4/44	Crimi et al., 2013
Retrospective	Diabetes (yes/no)	—	RIC	6/65	Sloth et al., 2015
Retrospective	Pre‐infarction angina (yes/no)	—	RIC	54/55	Pryds, Bøttcher, et al., 2016
Co‐medications					
Retrospective	ACE inhibitors (yes/no)	—	RIC	14/55	Sloth et al., 2015
Retrospective	ARBs(yes/no)	—	RIC	10/59	Sloth et al., 2015
Retrospective	β‐blockers (yes/no)	—	RIC	11/58	Sloth et al., 2015
Retrospective	Calcium channel blockers (yes/no)	—	RIC	7/62	Sloth et al., 2015
Retrospective	Statins (yes/no)	↑^a^	RIC	12/59	Sloth et al., 2015
Retrospective	Opioids (yes/no)	—	RIC	26/22	Crimi et al., 2013
Prospective	Morphine (yes/no)	—	RIC	33/33	Rentoukas et al., 2010
Pre‐specified	Glycoprotein IIb/IIIa inhibitors (yes/no)	—	RIC + PoCo	57/175	Eitel et al., 2015
Peri‐procedural determinants					
Prospective	Collateral perfusion/collateral blood flow	—	IPC	18/18	Argaud et al., 2004
Retrospective	Collateral perfusion/collateral blood flow	—	RIC	43/40	Pryds, Bøttcher, et al., 2016
Retrospective	Duration of chest pain (<5 hr)	↓	RIC	38/28	Pryds, Bøttcher, et al., 2016
Pre‐specified	Direct stenting (yes/no)	—	RIC + PoCo	170/62	Eitel et al., 2015
Pre‐specified	thrombectomy (yes/no)	—	RIC + PoCo	152/80	Eitel et al., 2015
Retrospective	Thrombectomy (yes/no)	↓^a^	PoCo	326/291	Nepper‐Christensen et al., 2019
Cardiovascular surgery					
Risk factors/co‐morbidities					
Retrospective	Age (≤63/64–72/ ≥73 years)	—	RIC	59/49/54	Kleinbongard et al., 2016
Retrospective	Sex (male/female)	—	RIC	269/60	Kleinbongard et al., 2016
Exploratory	Diabetes (yes/no)	—	RIC	39/29	Kottenberg, Thielmann, et al., 2014
Co‐medications					
Meta‐analysis	β‐blockers (yes/no)	↓	RIC	1155 (total)	Zhou et al., 2013
Retrospective	β‐blockers (yes/no)	—	RIC	227/102	Kleinbongard et al., 2016
Retrospective	Sulphonylureas (yes/no)	↓	RIC	16/100	Kottenberg, Thielmann, et al., 2014
Peri‐procedural determinants					
Prospective	Propofol/isoflurane	↓	RIC	14/20	Kottenberg et al., 2012
Meta‐analysis	Propofol/volatile anaesthesia	↓	RIC	902/751	Zangrillo et al., 2015
Prospective	Nitroglycerin (yes/no)	↓	RIC	53/36	Candilio et al., 2015
Retrospective	Nitroglycerin (yes/no)	—	RIC	16/20	Kleinbongard et al., 2013
Retrospective	Cross‐clamp time (≤56 min)	↓	RIC	50/59/53	Kleinbongard et al., 2016

Abbreviations: ACE, angiotensin‐converting enzyme; ARBs, AT_1_ receptor blockers ; IPC, local ischaemic preconditioning; PoCo, local ischaemic postconditioning; RIC, remote ischaemic conditioning.

a Non‐significant effect.

## 2 CONFOUNDERS OF CARDIOPROTECTION IN ACUTE MYOCARDIAL INFARCTION

Proof‐of‐concept studies using pharmacological approaches with cyclosporine (Piot et al., 2008) and exenatide (Lonborg et al., 2012a) and mechanical approaches by local ischaemic post‐conditioning and remote ischaemic conditioning (RIC) have documented the potential of attenuating ischaemia/reperfusion injury (see Figures S1 and S2) in terms of reducing biomarker release or infarct size (Bøtker, Lassen, & Jespersen, 2018; Heusch & Rassaf, 2016). However, in larger phase III trials, neither cyclosporine nor exenatide was confirmed to reduce infarct size and improve clinical outcome (Cung et al., 2015; Engstrøm et al., 2017).

### 2.1 INFARCT SIZE AND LOCATION

In clinical practice, infarct size following ST‐segment elevation myocardial infarction (STEMI) varies widely depending on the location of the coronary occlusion, ischaemia duration, collateral blood flow, and spontaneous recanalization. Reperfusion per se can reduce median infarct size to ≤50% of the area at risk (Bøtker et al., 2010; Kaltoft et al., 2006). Average median infarct size with modern reperfusion therapy and admission logistics is 15% of the left ventricle (LV). No definitive threshold of infarct size for increasing mortality (Burns et al., 2002) or reducing left ventricular ejection fraction (LVEF; Burns et al., 2002; Tarantini et al., 2006) has been established. The 6‐month mortality rate is approximately 2% in patients with infarct sizes larger than 20% of the LV, increasing to 4.5% at infarct sizes more than 35% of the LV. An infarct size ≤12% is associated with very low mortality and morbidity (Kastrati et al., 2002; Schömig et al., 2004). Hence, achievement of the smallest possible infarct size should always be aimed for, and this goal justifies cardioprotective therapy beyond revascularization. However, with the improved prognosis experienced over the last 25 years (Schmidt, Jacobsen, Lash, Bøtker, & Sørensen, 2012), translation of infarct size reduction into improved clinical outcome by any cardioprotective therapy is challenging, especially at low infarct sizes. Because clinical outcome is only significantly compromised when more than 20% of the LV is infarcted, prevention of mortality and heart failure in trials evaluating cardioprotective therapy beyond revascularization can only be documented in patients with large areas at risk and a potential for infarct size reduction to <20% of the LV. These patients, predominantly suffering from large anterior infarcts after proximal left anterior descending coronary artery occlusion, only constitute a quarter of all STEMI patients who allow measurable clinical benefit from adjunctive therapy in clinical trials.

Patients presenting with right and/or circumflex coronary artery occlusion have relatively small infarct sizes. Hence, myocardial salvage is minor, the clinical outcome is already fair, and the benefit of cardioprotective therapy not as much as in those presenting with proximal left anterior descending coronary artery occlusion, where the infarct size is significantly larger (Bøtker et al., 2010; Piot et al., 2008). “All‐comer” trials will lead to the recruitment of many patients with small infarcts and little additional myocardial salvage, which may dilute the positive effect elicited by any novel protective strategy. Restricting recruitment to patients with large anterior infarcts limits the external validity of a clinical study. A valid approach is to include a sufficient number of patients with anterior infarcts, who have the most severe clinical outcome. Such approach requires fewer patients, such that the study is powered to detect a clinical benefit in those with the most severe outcome (Hausenloy et al., 2013; Hausenloy et al., 2015). Of note, a recent study by Gaspar et al demonstrated that infarct size reduction by RIC translates into improved clinical outcome in STEMI patients, particularly when LV function is compromised (Gaspar et al., 2018; Heusch, 2018).

### 2.2 TIMI FLOW PRIOR TO REVASCULARIZATION

Approximately 40% patients presenting with STEMI have undergone spontaneous reperfusion prior to interventional reperfusion (Bøtker et al., 2010). Such patients benefit less from adjunct cardioprotective therapy than those with occluded vessels on admission to the hospital (Bøtker et al., 2010; Roubille et al., 2014). Consequently, the efficacy of adjunct cardioprotective therapy must be analysed by stratification according to thrombolysis in myocardial infarction (TIMI) 0–1 and ≥2.

### 2.3 EFFECTS OF CORONARY COLLATERALS

Coronary collateral flow modifies the size of an evolving myocardial infarction. In STEMI patients, substantial collateralization reduces the sizes of the area at risk and the evolving infarct. Although available evidence is equivocal (Lonborg et al., 2012b; Ortiz‐Perez et al., 2010), extensive collateralization may attenuate the ability to demonstrate an effect of any novel cardioprotective strategy (Desch et al., 2010; Ortiz‐Perez et al., 2010; Ovize, Thibault, & Przyklenk, 2013). Local ischaemic preconditioning remains effective during repeated brief coronary artery occlusion in the human heart (Argaud et al., 2004). Also, RIC is not associated with an increase in coronary collateral prevalence, suggesting that RIC does not confer cardioprotection through coronary collateral recruitment (Pryds, Bøttcher, et al., 2016). Even though a cardioprotective signal may be mediated in the absence of, or with very little, collateral flow (Crimi et al., 2013; Prunier et al., 2014; White et al., 2015), the coronary collateral circulation may be important for the distribution of pharmacological cardioprotective agents and circulating cardioprotective mediators underlying the effect of RIC in humans (Pryds, Bøttcher, et al., 2016). As a consequence of these equivocal findings, the efficacy of adjunct cardioprotective therapy must be assessed by stratified analysis using the Rentrop classification of visible collaterals for grade 0–1 versus ≥2.

### 2.4 DURATION OF CHEST PAIN, TIMING OF INTERVENTION, AND PRE‐INFARCTION ANGINA

Patients presenting with STEMI are recommended for interventional or thrombolytic reperfusion within 12 hr of the onset of chest pain (Ibanez et al., 2017) even though myocardial salvage can be obtained beyond the 12‐hr limit, also when the infarct‐related artery is totally occluded (Busk et al., 2009; Schömig et al., 2005). Because the crucial events of ischaemia/reperfusion injury occur during ischaemia and in the first few minutes of reperfusion, any pharmacological or mechanical cardioprotective strategy must be applied prior to opening the infarct‐related coronary artery. Early presentation and revascularization may lead to small myocardial infarcts, such that the patient will have little advantage from adjunctive therapy. With late presentation, on the other hand, infarct progress may have been finalized and the patient may derive little benefit from either intervention or an adjunct to reperfusion. A post hoc exploratory study of the cardioprotective effect of exenatide has demonstrated that this compound reduces infarct size predominantly in patients with short system delays (Lonborg et al., 2012b). In contrast, a post hoc exploratory analysis of the cardioprotective efficacy of RIC as an adjunct to primary percutaneous coronary intervention (PCI) demonstrated that while health care system delay was negatively associated with myocardial salvage in patients treated with primary PCI alone, this association was not present in patients treated with RIC, as the adjunct cardioprotective effect of RIC increased with extended health care system delay up to 5 hr (Pryds, Terkelsen et al., 2016). Prehospital administration of RIC, as used in that study, is not a requisite for achievement of a cardioprotective effect in patients with STEMI. Application of RIC may be effective even when initiated at the time of arrival in the primary PCI centre (Prunier et al., 2014; White et al., 2015). The optimum timing for adjunctive therapies to demonstrate maximal benefit is probably between 3 and 8 hr from time of symptom onset to time of reperfusion. Because RIC might involve systemic non‐cardiac protective mechanisms, its efficacy might depend not only on its timing with respect to reperfusion but also with respect to the onset of symptoms. RIC may be a potential adjunctive treatment strategy for patients with STEMI challenged by long health care system delays.

Pre‐infarction angina may be cardioprotective and improve survival in patients with acute myocardial infarction (Herrett et al., 2014; Schmidt, Horvath‐Puho, Pedersen, Sørensen, & Bøtker, 2015), but available evidence is inconsistent (Pryds, Bøttcher, et al., 2016). Potential underlying mechanisms are not only the development of coronary collaterals (Zimarino, D'Andreamatteo, Waksman, Epstein, & De Caterina, 2014) but also the activation of an inherent ischaemic preconditioning‐like effect (Heusch, 2001; Kloner et al., 1995), which may vary depending on the timing of pre‐infarction angina. Development of functional collateral vessels requires time (Zimarino et al., 2014), while a preconditioning effect should be immediate (Heusch, Bøtker, Przyklenk, Redington, & Yellon, 2015). Consistent with this assumption, patients with unstable angina or pre‐infarction angina closely preceding the acute myocardial infarction seem to have the most pronounced benefit in terms of mortality reduction (Herrett et al., 2014; Schmidt et al., 2015). While unknown for pharmacological cardioprotection, pre‐infarction angina prior to STEMI does not compromise the efficacy of RIC (Crimi et al., 2013; Eitel et al., 2015; Pryds, Bøttcher, et al., 2016).

### 2.5 RISK FACTORS

Ageing, sex, obesity, and smoking are cardiovascular risk factors that are known to modify the efficacy of cardioprotective strategies in experimental settings (Ferdinandy et al., 2014). A major limitation of studies in humans is that the assessment of interaction between risk factors and the efficacy of cardioprotective strategies relies on surrogate markers of cardioprotection and retrospective or post hoc analyses.

In humans, ageing attenuates the efficacy of cardioprotection. Using endothelial function as a surrogate marker of cardioprotection (Kharbanda et al., 2002), ischaemic preconditioning protected against ischaemia/reperfusion‐induced endothelial dysfunction of the upper arm in healthy volunteers in their 20s but not in of men aged 68 years and above (Moro, Pedone, Mondi, Nunziata, & Antonelli Incalzi, 2011; van den Munckhof et al., 2013). Specifically in patients undergoing primary PCI, post‐conditioning by multiple balloon inflations failed to reduce irreversible injury, as evaluated by creatine kinase release, in patients above the age of 65 years (Darling, Solari, Smith, Furman, & Przyklenk, 2007). Using improvement of LV function following primary PCI as endpoint, a meta‐analysis implied a beneficial effect of postconditioning in patients younger than 62 years only (Zhou et al., 2012). In contrast, a post hoc and pre‐specified analysis of a limited number of STEMI patients undergoing primary PCI demonstrated no attenuation of the efficacy of RIC as evaluated by myocardial salvage index in single‐photon emission CT (SPECT; Sloth et al., 2015).

In accordance with experimental experience, female hearts have an increased resistance to ischaemia/reperfusion injury among patients undergoing primary PCI (Canali et al., 2012). RIC as an adjunct to primary PCI can increase the inherent high tolerance of the adult female heart further, such that sex does not compromise the efficacy for increasing myocardial salvage in females compared to males (Crimi et al., 2013; Eitel et al., 2015; Sloth et al., 2015).

Any influence of obesity on ischaemia/reperfusion injury is thought to be associated with obesity‐related insulin resistance. The information about body mass index, infarct size, and its association with clinical outcome is discrepant (for review, see Ferdinandy et al., 2014), and obesity is not unequivocally associated with compromised clinical outcome, hence the concept of the “obesity paradox.” In patients undergoing primary PCI, the efficacy of adjunctive RIC did not differ between patients with a body mass index <25 and ≥25 kg·m^−2^ (Sloth et al., 2015).

The detrimental effects of smoking on the cardiovascular system, such as endothelial dysfunction and activation of systemic inflammatory and prothrombotic processes, are mediated through a complex interaction of several chemical compounds in tobacco smoke (Messner & Bernhard, 2014). In a post hoc analysis of RIC in patients undergoing primary PCI, smoking attenuated the efficacy of the adjunctive cardioprotective treatment, albeit this effect was not statistically significant (Sloth et al., 2015). Smoking may disrupt some of the transduction pathways involved in RIC, and such interaction should be a subject for further investigation in experimental and clinical studies.

### 2.6 COMORBIDITIES

#### 2.6.1 Dyslipidaemia

In most preclinical studies, hyperlipidaemia worsens the outcome of ischaemia/reperfusion injury and attenuates the cardioprotective effect of both early and late preconditioning, postconditioning, and pharmacological conditioning. The underlying mechanisms involve hyperlipidaemia‐induced changes in cardioprotective signalling pathways. Clinical studies in STEMI patients have demonstrated that RIC was effective in hyperlipidaemic patients (Sloth et al., 2015). However, further studies are required to clarify whether adaptive cardioprotective mechanisms can be enhanced in hyperlipidaemic patients.

#### 2.6.2 Diabetes mellitus

Whereas preclinical studies have shown divergent results with respect to infarct size dependent on animal model and diabetes duration, the majority of clinical studies demonstrate worse outcomes from acute myocardial infarction in diabetic patients (Ferdinandy et al., 2014). Data in humans have confirmed that the resistance to the protective effect of ischaemic preconditioning, seen in many but not all experimental studies, may translate to patients (Engbersen et al., 2012), but this interaction is of minor importance in the clinical setting of STEMI because preconditioning is not applicable in unpredictable ischaemia. Of note, no clinical trials have investigated the effect of diabetes on the cardioprotective efficacy of pharmacological or ischaemic postconditioning in STEMI patients.

In a post hoc study of 71 patients treated with RIC prior to primary PCI, there was no difference in myocardial salvage index compared to 68 patients undergoing primary PCI alone (Sloth et al., 2015). Only six (8%) and eight (12%) patients, respectively, had diabetes mellitus and were on oral anti‐diabetic treatment, the majority with metformin. The increment of myocardial salvage index by RIC was not significantly lower in patients with than in those without diabetes. The findings were confirmed using biochemical marker release (Crimi et al., 2013). Even though the latter was a pre‐specified analysis, both studies have power limitations. In a randomized study including 200 elderly patients with diabetes mellitus undergoing elective PCI, RIC did not significantly reduce peri‐procedural myocardial injury (Xu et al., 2014). However, the authors did not demonstrate that their RIC protocol was effective in younger patients without diabetes either.

Two translational studies have shown the complexity of cardioprotection in humans with diabetes mellitus. The first study demonstrated that the effect of RIC is dependent on preserved neural pathways in patients with diabetes mellitus (Jensen, Stottrup, Kristiansen, & Bøtker, 2012). The cardioprotective efficacy of the humoral factor was tested in an isolated perfused rabbit heart ischaemia/reperfusion model. Therefore, the study could not clarify whether the diabetic heart itself was responsive to cardioprotection by RIC. In a subsequent study (Jensen et al., 2013), human right atrial trabeculae from diabetic patients had increased post‐ischaemic contractile function compared to trabeculae from patients without diabetes. Plasma dialysate harvested from either normal volunteers or diabetic patients treated with RIC protected non‐diabetic but not diabetic human right atrial trabeculae subjected to simulated ischaemia/reperfusion injury. The findings were associated with increased levels of myocardial O‐linked b‐*N*‐acetylglucosamine (O‐GlcNAc), suggesting that increased myocardial levels of O‐GlcNAc reflect a cardioprotective phenotype. In fact, diabetes mellitus appears to be associated with an inherent cardioprotective phenotype that may attenuate further protection by an external stimulus such as RIC. The clinical impact of diabetes mellitus on the efficacy of cardioprotective strategies requires certainly further investigation.

#### 2.6.3 Hypertension and LV hypertrophy

While protection remains present in animals with hypertension and/or left ventricular hypertrophy following preconditioning and infarct size reduction by ischaemic postconditioning appears to be lost (Ferdinandy et al., 2014), clinical data are sparse. In a study evaluating the effect of RIC on flow‐mediated vasodilation in the elderly, the increase in vasodilation after RIC was greater in the healthy elderly than in elderly patients with hypertension (Moro et al., 2011). A post hoc analysis of STEMI patients undergoing RIC before revascularization included very few patients with LV hypertrophy, in whom the effect of RIC was statistically insignificantly reduced, while in patients with hypertension, the effect of RIC was preserved (Sloth et al., 2015). This study did not take the duration of hypertension into account. The effects of LV hypertrophy for adjunctive cardioprotective strategies in the clinic still needs clarification.

Kidney failure is associated with a high prevalence of ischaemic heart disease; uraemia is a complex metabolic disease and could therefore potentially interfere with cardioprotection. However, currently, there are no clinical data available on this.

A word of caution is necessary when interpreting this clinical information. Most of these analyses are univariate and do not consider that patients very often carry several co‐morbidities. Intriguingly, neither Killip class nor cardiac arrest at the time of STEMI presentation seems to influence the effect of RIC (Eitel et al., 2015; Ladejobi et al., 2017).

### 2.7 CO‐MEDICATIONS

As demonstrated in a number of experimental studies, pharmacological therapy may alter the effects of cardioprotection.

ACE inhibitors and ARBs lower the threshold to achieve endogenous cardioprotection, especially in hearts with comorbidities, in experimental settings. In STEMI patients, the efficacy of RIC was preserved by such co‐treatment (Sloth et al., 2015).

#### 2.7.1 β‐blockers

Although the cardioprotective effect of post‐myocardial infarction treatment with β‐blockers in the era before revascularization is well documented, the efficacy of long‐term post‐infarction treatment in the revascularization era is questioned because the beneficial clinical effect is mainly seen within a few months following the infarct (Bangalore et al., 2014). Early intravenous metoprolol before reperfusion may reduce infarct size (Ibanez et al., 2013; Roolvink et al., 2016). In STEMI patients, the efficacy of RIC was preserved in β‐blocker users (Sloth et al., 2015). Given the widespread use of β‐blocker therapy, further studies and more detailed analyses are needed to clarify their interaction with other cardioprotective strategies.

#### 2.7.2 Calcium channel blockers

Clinical trials in acute myocardial infarction have not demonstrated significant infarct size reduction by either 1,4‐dihydropyridines, verapamil, or diltiazem (Opie, Yusuf, & Kübler, 2000), probably because the agents need to be given before the onset of ischaemia or during the early ischaemic phase. In STEMI patients, the efficacy of RIC was not affected by concomitant calcium channel blocker treatment (Sloth et al., 2015).

#### 2.7.3 Nitrates

Preclinical studies indicate that the presence of nitrate tolerance aggravates ischaemia/reperfusion injury and leads to loss of the cardioprotective effect of preconditioning (Ferdinandy et al., 2014; Gori et al., 2010). Also, the preconditioning effect of a single dose of nitroglycerin on endothelial function is lost upon a prolonged exposure to nitroglycerin in humans. Most recently, a translational study in rats and humans demonstrated that combined RIC and long‐term nitroglycerin treatment abolishes their individual protective effects on ischaemia/reperfusion injury, suggesting an interaction of clinical importance (Hauerslev et al., 2018). At present, no studies have been performed to clarify whether nitroglycerin interferes with the efficacy of cardioprotection by ischaemic conditioning in STEMI patients undergoing acute revascularization. A retrospective subgroup analysis of a study on cardioprotection by inhaled nitric oxide in STEMI patients identified an interaction of inhaled NO and intra‐arterial nitroglycerin. Nitroglycerin‐naïve patients had reduced infarct size by inhaled NO, those with nitroglycerin not (Janssens, Bogaert et al., 2018). In a registry, chronic nitrate treatment of STEMI patients was associated with less cardiac biomarker release, indicating cardioprotection by chronic nitrate treatment per se (Ambrosio et al., 2010). However, there are no data available on acute nitrate treatment and cardioprotection in STEMI patients.

#### 2.7.4 Opioids

Morphine and limb RIC protect against myocardial ischaemia/reperfusion injury in an experimental setting (Wang et al., 2016). A single‐centre, parallel‐group, randomized study of STEMI patients undergoing primary PCI also demonstrated a cardioprotective effect of RIC and morphine (Rentoukas et al., 2010). A combination of morphine and RIC did not confer a statistically significant improvement of the primary endpoint (full ST‐segment resolution) but was associated with larger ST‐segment deviation score reduction and lower peak troponin I levels. As, in this study, the effect of morphine alone was not assessed, it remains unclear whether morphine simply induced cardioprotection per se and added to the effect of RIC or whether morphine facilitated the efficacy of RIC. Crimi et al. (2013) did not observe any interaction between the efficacy of RIC and morphine. A post hoc analysis of the CIRCUS trial revealed that morphine neither reduced infarct size nor limited the incidence of MACE in anterior STEMI patients (Bonin et al., 2018). In experiments, RIC was blocked by pretreatment with the opioid receptor blocker naloxone (Shimizu et al., 2009). In a translational study using transfer of human dialysate to a rabbit heart, the infarct reducing effect of RIC was also blocked by naloxone (Michelsen et al., 2012). These findings suggest that an opioid action is involved in the mechanisms underlying RIC but addition of morphine treatment to RIC does not attenuate the cardioprotective capacity. Opioids are most frequently administered prior to the onset of reperfusion in clinical practice, and therefore, their potential interaction with cardioprotective interventions is important.

#### 2.7.5 Anti‐platelet agents

Although the clinical benefit of anti‐platelet drugs has been attributed to their anti‐thrombotic action, experimental and clinical data suggest that some of the anti‐platelet agents may also reduce infarct size. In an early retrospective analysis of a well‐selected cohort of STEMI patients, who were consecutively entered into clinical trials evaluating the influence of ischaemic or pharmacological postconditioning on infarct size, clopidogrel independently attenuated lethal reperfusion injury, as determined by cardiac biomarker release (Roubille et al., 2012). The protective effect appeared to add to the benefit afforded by ischaemic postconditioning. Animal data also suggest that the P2Y_12_ receptor antagonists ticagrelor and cangrelor have pleiotropic effects, potentially through adenosine‐like and anti‐inflammatory effects, that afford even greater protection against tissue injury than clopidogrel (Adamski et al., 2014; Cohen & Downey, 2017; Nanhwan et al., 2014; Yang et al., 2012). The protective effect has been translated to humans using endothelial function as endpoint (Weisshaar, Litschauer, Eipeldauer, Hobl, & Wolzt, 2017). It still remains unknown to what extent a direct cardioprotective effect of ticagrelor is involved in the mechanisms underlying its superior effect over that of clopidogrel in patients with acute myocardial infarction (Wallentin et al., 2009), including those with or without ST elevation who undergo planned revascularization (Cannon et al., 2010). While RIC increases myocardial salvage above any protection afforded by clopidogrel (Bøtker et al., 2010), it is unknown whether the conditioning‐mimetic effect of ticagrelor and cangrelor, shown to be efficacious in animal models, may be a confounding factor in clinical trials.

Stone et al. (2012) reported that intracoronary abciximab significantly reduced infarct size in STEMI patients. Administration of glycoprotein IIb/IIIa inhibitors at the time of primary PCI, the use of which is declining in clinical practice, does not seem to compromise myocardial salvage from RIC (Eitel et al., 2015).

#### 2.7.6 Statins and anti‐hyperlipidaemic drugs

Statins protect the heart against ischaemia/reperfusion injury in preclinical studies, but statin treatment may interfere with the infarct size‐limiting effect of preconditioning (Ferdinandy et al., 2014). Because many, but not all, clinical trials have shown efficacy in reducing major coronary adverse effects, including mortality, when high‐dose statins were administered before PCI (elective or in patients with non‐ST elevation acute coronary syndromes), guidelines have previously recommended high‐dose statins for patients with STEMI and non‐STEMI. However, the efficacy of statin treatment to reduce myocardial infarct size, as assessed by cardiac magnetic resonance imaging and SPECT, in patients presenting with STEMI is equivocal (Birnbaum, Birnbaum, Ye, & Birnbaum, 2015). In clinical trials, statins have been added to a standard‐of‐care regimen, which includes several medications that open the possibility of drug interactions, such that the outcome may differ from the experimental situation and may also differ depending on the statin therapy initiated in a high dose just prior to or given as long‐term chronic therapy ahead of revascularization. In a post hoc analysis, the efficacy of RIC was more pronounced in long‐term statin users prior to primary PCI (Sloth, Schmidt et al., 2015). However, this finding and the potential effect of drug interactions influencing the efficacy of cardioprotection need further investigation.

#### 2.7.7 Anti‐diabetic treatment

Specific sulphonylureas used to treat Type 2 diabetes can attenuate the conditioning response not only in experimental (Kristiansen et al., 2011) but also in clinical settings (Kottenberg, Thielmann, et al., 2014). Glyburide (glibenclamide) blocks the ATP‐sensitive K channels and the protective effects of ischaemic and various forms of pharmacological preconditioning and postconditioning (Birnbaum et al., 2015; Ye, Perez‐Polo, Aguilar, & Birnbaum, 2011). Conversely, insulin, metformin, glucagon‐like peptide‐1 (GLP‐1) analogues, gliptins (dipeptidyl peptidase‐4 [DPP‐4] inhibitors that prevent degradation of GLP‐1), and sodium glucose transporter‐2 (SGLT‐2) inhibitors may be cardioprotective per se and may either raise the threshold for an additional benefit or act synergistically with other cardioprotective drugs and mechanical conditioning strategies.

Because hyperglycaemia is associated with poorer outcome in the setting of acute myocardial infarction, numerous studies have investigated the cardioprotective effect of insulin in patients with acute myocardial infarction with and without diabetes mellitus. To avoid hypoglycaemia, glucose has been given simultaneously. A glucose and insulin infusion causes potassium to move intracellularly, so the addition of exogenous potassium (glucose–insulin–potassium [GIK] infusion) helps to prevent hypokalaemia and to electrically stabilize the cardiomyocyte cell membrane to avoid arrhythmias. The studies were conducted in the pre‐revascularization era (Kloner & Nesto, 2008). Although a subgroup of patients with diabetes mellitus had an increase in myocardial salvage index (Pache et al., 2004), and in another study a lower increase in troponin levels with GIK (Yazici et al., 2005), these studies were exceptions. Most studies that assessed myocardial infarct size by SPECT or creatine kinase muscle–brain release failed to show a benefit of GIK. Consequently, the IMMEDIATE trial subsequently tested out‐of‐hospital emergency medical service administration of GIK in the first hours of suspected acute coronary syndromes (Selker et al., 2012). The study did not specifically examine a potential reduction of ischaemia/reperfusion injury. The study revealed no effect of GIK on 30‐day mortality in the entire cohort or among patients presenting with ST‐segment elevation undergoing immediate revascularization according to guidelines. However, the composite of cardiac arrest or in‐hospital mortality was reduced for those treated with GIK, both among all those with acute coronary syndromes and among those presenting with ST‐segment elevation. The interaction between insulin and other ischaemic or pharmacological conditioning strategies has not been studied in a clinical setting of STEMI patients undergoing revascularization.

Most clinical studies of metformin have been conducted in the pre‐revascularization era. Several (Roussel et al., 2010) but not all studies (Mellbin et al., 2008) have revealed a cardioprotective effect in terms of mortality, readmission for myocardial infarction and admission for heart failure (Varjabedian, Bourji, Pourafkari, & Nader, 2018). In a retrospective study, chronic metformin treatment of diabetic patients prior to STEMI was associated with reduced biomarker release (Lexis et al., 2014). However, another retrospective study did not confirm such benefit (Basnet et al., 2015). The acute effect of metformin on ischaemia/reperfusion injury has not been clarified in a clinical setting. Non‐diabetic STEMI patients receiving metformin with the onset of reperfusion for 4 months had no improvement of LVEF (Lexis et al., 2014). Although metformin has been shown to inhibit mitochondrial permeability transition pore opening in vitro (Guigas et al., 2004), it is also unclear whether metformin has interactions that augment or inhibit the effects of other ischaemic or pharmacological conditioning strategies.

Two clinical trials have shown that exenatide, a GLP‐1 analogue, limits infarct size in patients with STEMI undergoing primary PCI (Lonborg et al., 2012b; Woo et al., 2013), whereas two other trials failed to show reduction of cardiovascular events with long‐term DPP‐4 inhibitor treatment (Scirica et al., 2013; White et al., 2013). The outcome of combination therapies is unknown, but an ongoing trial is assessing whether RIC and intravenous exenatide immediately before primary PCI reduce infarct size measured by cardiac MRI performed 3–7 days after primary PCI (NCT02404376).

Recent experimental data indicate that SGLT‐2 inhibitors reduce infarct size irrespective of diabetic status (Baker et al., 2019; Lim et al., 2019). SGLT‐2 inhibitors reduce heart failure and cardiovascular mortality in patients with Type 2 diabetes mellitus in the absence of a simultaneous reduction in the incidence of nonfatal myocardial infarction or nonfatal stroke (Neal et al., 2017; Wiviott et al., 2018; Zinman et al., 2015). Hence, the benefit of SGLT‐2 inhibitors appears to be caused by improvement in patients, who experience a cardiovascular event rather than in the prevention of atherosclerotic events, thus suggesting cardioprotection. Further studies are required to confirm this hypothesis.

There may also be an unappreciated interaction between co‐morbidities and co‐medications. In an experimental mouse model of Type 2 diabetes/metabolic syndrome with hypoxic preconditioning, treatment of one comorbidity (hypertension or heart failure with ACE inhibition) restored the contractile response which had been compromised by another co‐morbidity (diabetes; Van der Mieren et al., 2013).

### 2.8 PROCEDURE‐RELATED TREATMENT

#### 2.8.1 Stenting technique

Direct stenting allows immediate full reperfusion without any interference by a residual stenosis or by coronary microembolization (Loubeyre et al., 2002). Consequently, direct stenting may be preferable to obtain maximum yield of postconditioning (Heusch, 2012).

#### 2.8.2 Thrombectomy

Routine thrombectomy does not yield cardioprotection (Kaltoft et al., 2006) or improvement of outcome in patients undergoing primary PCI (Lagerqvist et al., 2014). Thrombectomy and direct stenting do not seem to influence the cardioprotective potential of RIC (Eitel et al., 2015). However, thrombectomy interfered with ischaemic postconditioning in the DANAMI‐3 ipost trial; patients with STEMI undergoing primary PCI with thrombectomy were not protected by ischaemic postconditioning whereas patients undergoing primary PCI without thrombectomy had better clinical outcome (reduced all‐cause mortality and hospitalization for heart failure over a median follow‐up of 35 months), suggesting that thrombectomy had delayed the postconditioning procedure and/or caused coronary microembolization (Nepper‐Christensen et al., 2019). Exactly such interference of coronary microembolization with reduction of infarct size by ischaemic postconditioning has previously been reported in a pig model of myocardial infarction (Skyschally, Walter, & Heusch, 2013).

## 3 CONFOUNDERS OF CARDIOPROTECTION IN CARDIOVASCULAR SURGERY

Coronary artery bypass graft (CABG) surgery remains the most frequent cardiovascular surgery, followed by isolated aortic valve replacement, combined CABG surgery and aortic valve replacement, mitral valve replacement and operations for aortic aneurysms. Due to an ageing and increasingly co‐morbid population, there is a growing need for combined surgical procedures. Post‐operative mortality is highest in combined surgery and lowest for isolated mitral valve replacement (Adelborg et al., 2017). Most anaesthetics reduce myocardial ischaemia/reperfusion damage per se (Kleinbongard & Heusch, 2015; Zaugg, Lucchinetti, Behmanesh, & Clanachan, 2014). Initially, a superior benefit from volatile anaesthetics was discussed (Straarup, Hausenloy, & Rolighed Larsen, 2016; Symons & Myles, 2006), but in a recent multi‐centre trial, volatile anaesthetics and total intravenous anaesthesia were comparable in terms of 1‐year mortality (Landoni et al., 2019). Most cardiovascular surgeries are performed under protection by cardioplegic arrest, and protection by blood and crystalloid cardioplegia is comparable in outcome (Guru, Omura, Alghamdi, Weisel, & Fremes, 2006). Also, CABG surgery versus off‐pump surgery on a beating heart do not differ in 5‐year clinical outcome (Lamy et al., 2016; Shroyer et al., 2017).

Cardiovascular surgery under cardioplegic ischaemic cardiac arrest results in perioperative myocardial ischaemia/reperfusion injury and an additional traumatic injury from surgical handling (Birdi, Angelini, & Bryan, 1997). Obviously, valve surgery causes more traumatic injury than CABG surgery (D'Agostino et al., 2019). Elevation in cardiac biomarkers (creatine kinase, creatine kinase muscle–brain, and troponin) post‐surgery is used to quantify myocardial injury. Such biomarker release, however, reflects both, the ischaemia/reperfusion‐induced and the traumatic injury. Biomarker release correlates with imaging parameters for myocardial viability (late gadolinium enhancement in cardiovascular magnetic resonance); however, the prognostic significance of biomarker release remains to be determined (Thielmann et al., 2017).

Patients undergoing cardiovascular surgery are mainly male and elderly and have diabetes, vascular disease, chronic obstructive pulmonary disease, or renal dysfunction. These co‐morbidities affect the clinical outcome per se. However, in recent studies, the 10‐year clinical outcome was similar between the sexes after risk adjustment (den Ruijter et al., 2015; Pina et al., 2018). As observed with acute myocardial infarction, patients with angina prior to cardiovascular surgery have lower all‐cause mortality over a follow‐up time of about 4.5 years (Jolicoeur et al., 2015).

Most patients undergoing cardiovascular surgery are on baseline medications such as ACE inhibitors or ARBs, β‐blockers, nitroglycerin, and statins (Sousa‐Uva et al., 2018), all of which induce cardioprotection per se (see above).

The interaction between co‐morbidities and co‐medications and cardioprotection in cardiovascular surgery is not really different from that in acute myocardial infarction. A number of smaller studies in patients undergoing cardiovascular surgery have reported cardioprotection by mechanical approaches such as local ischaemic preconditioning, postconditioning, and RIC as well as by pharmacological approaches (see Figures S3 and S4). However, there is no cardioprotective approach with an unequivocal impact on the release of biomarkers or, more importantly, clinical outcome. The interaction between patient specific pre‐existing and/or additional intra‐operative confounders with the cardioprotective approaches may be one reason for the inconsistent data.

### 3.1 PRE‐EXISTING CONFOUNDERS

#### 3.1.1 Demographics

Ageing attenuates cardioprotection by conditioning strategies (Boengler et al., 2009; Ferdinandy, Hausenloy et al., 2014; Heusch, 2013). However, in a retrospective analysis of patients undergoing CABG surgery with/without RIC age did not interfere with cardioprotection. In fact, troponin release was reduced in patients ≤63, 64–72, and ≥73 years (Kleinbongard et al., 2016). RIC also induced cardioprotection, reflected by lower increase in troponin or creatine kinase muscle–brain, in children (see Figure S3).Experimental studies suggest a natural resistance of female hearts to ischaemia/xreperfusion injury (Ferdinandy et al., 2014). However, the retrospective analysis of RIC in patients undergoing CABG surgery did not identify sex as confounder; however, the number of females was small (Kleinbongard et al., 2016).

#### 3.1.2 Co‐morbidities

The interaction of co‐morbidities with cardioprotection in patients undergoing cardiovascular surgery is not well investigated. In one exploratory analysis, there was no reduction of troponin release by RIC in diabetic patients, irrespective of their anti‐diabetic drug treatment (Kottenberg, Thielmann, et al., 2014).

#### 3.1.3 Baseline co‐medications

Some medications recruit signalling steps of local and/or RIC (e.g., ACE inhibitors or ARBs, statins; Heusch, 2015; Kleinbongard & Heusch, 2015) and may thus interfere with cardioprotection. A meta‐analysis of 15 trials on RIC in cardiovascular surgery reported an attenuation of protection, as reflected by troponin or creatine kinase muscle–brain release, by RIC with β‐blockers (Zhou et al., 2013), but a retrospective analysis of a single‐centre trial did not identify such interaction (Kleinbongard et al., 2016). So far, there is no analysis of an interaction between protection by RIC and ACE inhibitors or ARBs (Kleinbongard et al., 2016; Sloth et al., 2015; Zhou et al., 2013). The reduction of troponin release by RIC was blocked in sulphonylurea‐treated diabetics (Kottenberg, Thielmann, et al., 2014).

### 3.2 INTRA‐OPERATIVE CONFOUNDERS

#### 3.2.1 Anaesthetics

Infarct size reduction by RIC was blocked when rats were anaesthetized with propofol (Cho et al., 2019), and this interference is also evident in humans. In prospective single‐centre trials, RIC decreased troponin release only in a smaller cohort of patients undergoing cardiac surgery under isoflurane (Kottenberg et al., 2012) or sevoflurane anaesthesia (Bautin et al., 2014; Bautin et al., 2015) but not under propofol anaesthesia. Increased signal transducer and activator of transcription 5 (STAT5) phosphorylation in the LV was associated with cardioprotection under isoflurane (Heusch et al., 2012) or sevoflurane (Wu et al., 2018) anaesthesia, but such increased STAT5 phosphorylation was absent under propofol (Kottenberg, Musiolik, et al., 2014). A Bayesian network meta‐analysis, of randomized trials, confirmed the interference: Combination of RIC with volatile anaesthesia, but not with total intravenous anaesthesia (propofol), reduced post‐operative mortality (Zangrillo et al., 2015).

#### 3.2.2 Cardioplegia

In patients undergoing CABG surgery with crystalloid cardioplegia (Thielmann et al., 2013) as well as those with blood cardioplegia (Hausenloy et al., 2007) RIC reduced troponin release.

#### 3.2.3 Cross‐clamp time

Obviously, ischaemia/reperfusion injury increases with longer cross‐clamp times; therefore, protection from such ischaemia/reperfusion injury becomes more evident at longer rather than shorter aortic cross‐clamp times. Along this line, pharmacologically induced reduction of troponin or creatine kinase muscle–brain release in patients undergoing CABG surgery by cyclosporine was seen with longer (85–120 min), but not with, shorter cross‐clamp times (50–85 min; Hausenloy et al., 2014). Also, RIC in patients undergoing CABG surgery did not reduce troponin release with an aortic cross‐clamp time ≤56 min but did so in those with 57–75 min (Kleinbongard et al., 2016). In patients undergoing aortic valve replacement with relatively short cross‐clamp time of about 55–59 min, there was no reduction of troponin release by RIC (Pinaud et al., 2015). On the other hand, cyclosporine also reduced troponin release in patients undergoing elective aortic valve surgery with a relatively short cross‐clamp time of about 54 min (Chiari et al., 2014).

#### 3.2.4 Types of heart surgery

Conditioning strategies only protect from ischaemia/reperfusion and not from traumatic injury. Consequently, when estimating myocardial injury via biomarker release, only part of this release can be reduced by conditioning approaches. RIC consistently reduced troponin release in patients undergoing elective CABG without valve surgery (Hausenloy et al., 2007; Thielmann et al., 2013). However, when cohorts comprised patients of ~50% combined CABG and valve surgery (Hausenloy et al., 2015) or 25% isolated valve surgery (Meybohm et al., 2015), RIC did not reduce troponin release. In contrast, RIC reduced troponin release in patients undergoing CABG and/or valve (36–39%) surgery (Candilio et al., 2015), and cyclosporine also reduced troponin release in those undergoing elective aortic valve surgery (Chiari et al., 2014).

#### 3.2.5 Intra‐operative medication

Nitroglycerin is administered during cardiovascular surgery to control blood pressure and dilate arterial grafts, and it exerts cardioprotection per se (Pagliaro, Gattullo, & Penna, 2013). In a prospective analysis, intra‐operatively administered intravenous nitroglycerin per se reduced troponin release and attenuated further cardioprotection by RIC (Candilio et al., 2015). In a retrospective analysis of another study, nitroglycerin did not interfere with protection, as determined from troponin release, by RIC (Kleinbongard, Thielmann et al., 2013). An ongoing prospectively designed, randomized controlled trial “Effect of RIC and Glyceryl Trinitrate on Perioperative Myocardial Injury in Cardiac Bypass Surgery Patients (ERIC‐GTN trial)” (Hamarneh et al., 2015) aims to resolve this controversy.

### 3.3 POST‐OPERATIVE CONFOUNDERS

Post‐operative drugs such as anti‐coagulants, anti‐arrhythmics, or inhibitors of the renin–angiotensin–aldosterone system attenuate LV dysfunction and/or and improve clinical outcome. However, the protection against ischaemia/reperfusion damage must occur at early reperfusion. Thus, such post‐operative treatment strategies can influence clinical outcome, not through reduction ischaemia/reperfusion damage, but through impairment of repair and attenuation of remodelling (Heusch et al., 2014).

## 4 CONCLUSIONS AND PERSPECTIVE

The clinical evidence for confounding of cardioprotection by risk factors, co‐morbidities, and co‐medications in patients suffering an acute myocardial infarction or undergoing cardiovascular surgery is much less robust than that obtained in experimental studies. One major difference between experimental and clinical studies is that patients suffer from multiple co‐morbidities and are treated by multiple medications whereas most animal models with co‐morbidities lack adequate treatment of the respective co‐morbidity. Otherwise, the clinical evidence is mostly derived from retrospective secondary analyses. These analyses mostly do not consider more than a single or a few potential confounders with appropriate risk‐adjusted, multivariate analysis. However, such analysis does also not exist for the experimental confounder studies. While a number of potential confounders were identified in the existing analyses, they certainly do not sufficiently explain the discrepancies between positive and neutral studies on infarct size in humans and the lack of translation to clinical benefit. The best clinical evidence for a confounder effect is to be found for anti‐platelet drugs which exert protection per se and thus limit the potential for further cardioprotection in patients with acute myocardial infarction. There is also clinical evidence for aortic cross‐clamp time duration to make the efficacy of protection more evident and for propofol to interfere with RIC in patients undergoing cardiovascular surgery (Figure 1). Finally, there are two important caveats: (a) The power of all clinical studies to detect a confounding effect of a single co‐morbidity or co‐medication given the multiple co‐morbidities and co‐medications of an individual patient is low, and (b) the number of clinical data available for the different settings of ischaemic and pharmacological conditioning is limited.

### 4.1 Nomenclature of targets and ligands

Key protein targets and ligands in this article are hyperlinked to corresponding entries in http://www.guidetopharmacology.org, the common portal for data from the IUPHAR/BPS Guide to PHARMACOLOGY (Harding et al., 2018), and are permanently archived in the Concise Guide to PHARMACOLOGY 2017/18 (Alexander, Christopoulos, et al., 2017; Alexander, Fabbro, et al., 2017a, b; Alexander, Kelly, et al., 2017).

## CONFLICT OF INTEREST

The authors declare no conflicts of interest.

## Supporting information


**Figure S1:**
Forest plot of clinical studies on ischaemic conditioning in patients with acute myocardial infarction and with biomarker release or imaging techniques to estimate infarct size as end‐point. The zero represents the mean value, and the gray bars represent the standard error of the mean for the placebo group. Closed squares represent significantly reduced infarct size (*x* ~ ± SEM), open squares represent non‐significant changes. CK(‐MB) = creatine kinase (muscle‐brain); IC = ischaemic conditioning group; MRI = magnetic resonance imaging; n.s. = not significant; PLA = placebo group; PoCo = local ischaemic postconditioning; RIC = remote ischaemic conditioning; SPECT = single‐photon emission computed tomography; Tn(I) = troponin (I);

**Figure S2:**
Forest plot of clinical studies on pharmacological conditioning in patients with acute myocardial infarction and with biomarker release or imaging techniques to estimate infarct size as end‐point. The zero represents the mean value, and the gray bars represent the standard error of the mean for the placebo group. Closed squares represent significantly reduced infarct size (*x* ~ ± SEM), open squares represent non‐significant changes. The star on the midline is used when no information was given to the standard error of the mean. ANP = atrial natriuretic peptide; CK(‐MB) = creatine kinase (muscle‐brain); drug = pharmacological conditioning group; Fx06 = peptide derived from the neo‐N‐terminus of fibrin; G‐CSF = granulocyte‐colony stimulating factor; GIK = glucose‐insulin‐potassium; (hs)Tn(I/T) = (high sensitive) troponin (I/T); IGF1 = insulin‐like growth factor 1; LeukArrest = rovelizumab; n.s. = not significant; MRI = magnetic resonance imaging; MTP‐131 = cardiolipin‐targeting peptide; PLA = placebo group; SNP = sodium nitroprusside; SPECT = single‐photon emission computed tomography; TRO40303 = 3,5‐seco‐4‐nor‐cholestan‐5‐one oxime‐3‐ol; * = dual anti platelet therapy

**Figure S3:**
Forest plot of clinical studies on ischaemic conditioning in patients undergoing cardiovascular surgery and with biomarker release as end‐point to estimate ischaemia/reperfusion injury. The zero represents the mean value, and the gray bars represent the standard error of the mean for the placebo group. Closed squares represent significantly reduced infarct size (*x* ~ ± SEM), open squares represent non‐significant changes. The star is used when no information was given to the standard error of the mean. CK‐MB = creatine kinase (muscle‐brain); (hs)Tn(I/T) = (high sensitive) troponin (I/T); ICU = propofol was given at the intensive care unit stay; IPC = local ischaemic preconditioning; n = no propofol anaesthesia; n.s. = not significant; PLA = placebo group; PoCo = local ischaemic postconditioning; RIC = remote ischaemic conditioning; RIC* = RIC in children undergoing cardiovascular surgery; RIC‐LT = repetitive long term RIC; y = yes, propofol anaesthesia;? = no information was given to the use of propofol

**Figure S4:**
Forest plot of clinical studies on pharmacological conditioning, on cardioprotection in patients undergoing cardiovascular surgery and with biomarker release as end‐point to estimate ischaemia/reperfusion injury. The zero represents the mean value, and the gray bars represent the standard error of the mean for the placebo group. Closed squares represent significantly reduced infarct size (*x* ~ ± SEM), open squares represent nonsignificant changes. The star on the midline is used when no information was given to the standard error of the mean. BNP = brain natriuretic peptide; CK‐MB = creatine kinase (muscle‐brain); GIK = glucose‐insulin‐potassium; GR79236X = adenosine receptor agonist; (hs)Tn(I/T) = (high sensitive) troponin (I/T); n = no propofol anaesthesia; n.s. = not significant; PLA = placebo group; y = yes, propofol anaesthesia; ? = no information was given to the use of propofol
