# Supplementary material for: Co‐morbidities and co‐medications as confounders of cardioprotection—Does it matter in the clinical setting?
Source: Br J Pharmacol. 2020 Jan 3;177(23):5252–69. doi: 10.1111/bph.14839 (PMC7680006; doi:10.1111/bph.14839)
Supplement: Supplementary file 1 — Figure S1: Forest plot of clinical studies on ischaemic conditioning in patients with acute myocardial infarction and with biomarker release or imaging techniques to estimate infarct size as end‐point. The zero represents the mean value, and the gray bars represent the standard error of the mean for the placebo group. Closed squares represent significantly reduced infarct size (x ~ ± SEM), open squares represent non‐significant changes. CK(‐MB) = creatine kinase (muscle‐brain); IC = ischaemic conditioning group; MRI = magnetic resonance imaging; n.s. = not significant; PLA = placebo group; PoCo = local ischaemic postconditioning; RIC = remote ischaemic conditioning; SPECT = single‐photon emission computed tomography; Tn(I) = troponin (I); [file BPH-177-5252-s001.pdf]

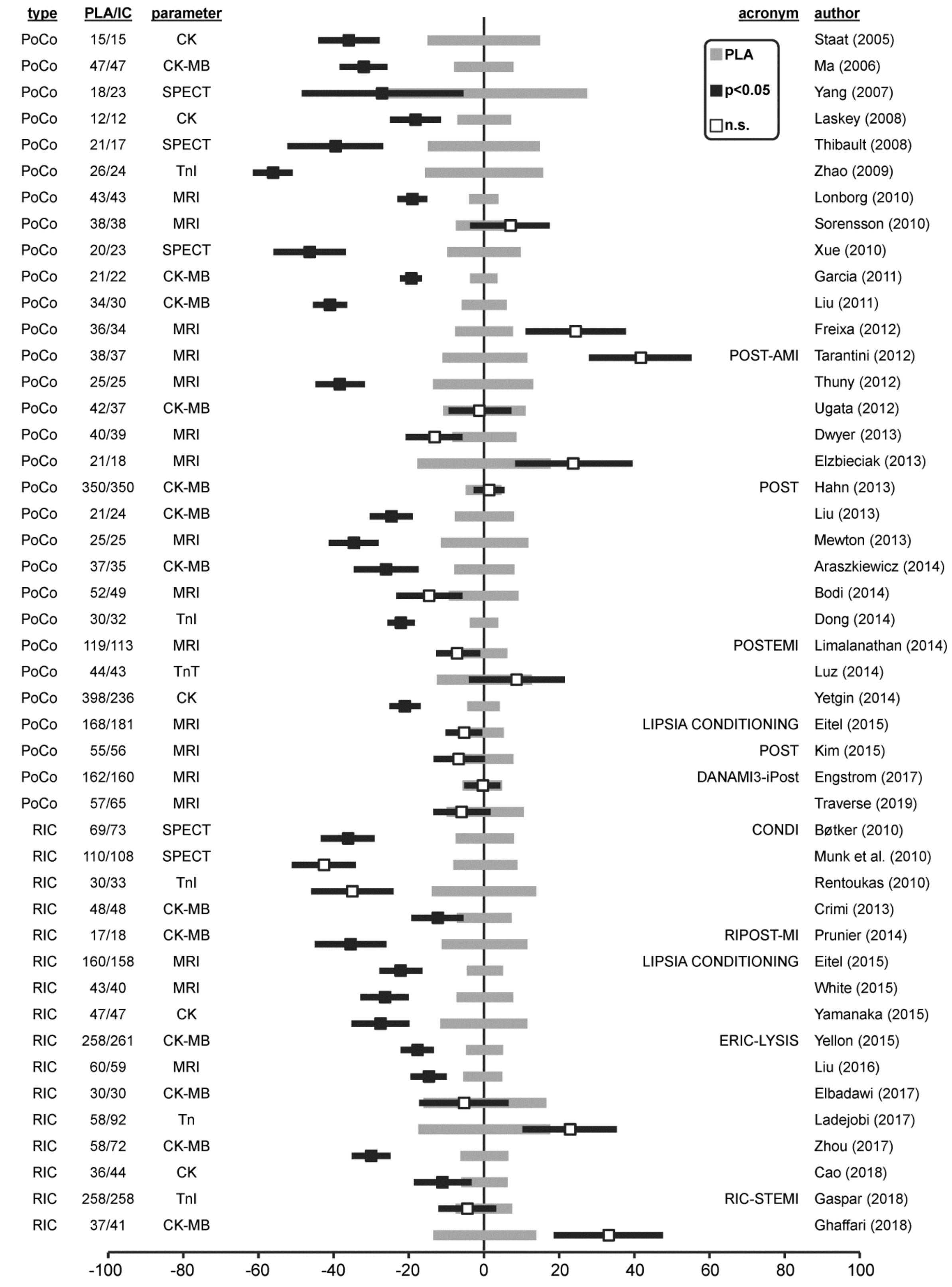

Supplemental Figure 1: Forest plot of clinical studies on ischaemic conditioning in patients with acute myocardial infarction and with biomarker release or imaging techniques to estimate infarct size as end-point. The zero represents the mean value, and the gray bars represent the standard error of the mean for the placebo group. Closed squares represent

significantly reduced infarct size ($\tilde{x}$ ±SEM), open squares represent non-significant changes. CK(-MB) = creatine kinase (muscle-brain); IC = ischaemic conditioning group; MRI = magnetic resonance imaging; n.s. = not significant; PLA = placebo group; PoCo = local ischaemic postconditioning; RIC = remote ischaemic conditioning; SPECT = single-photon emission computed tomography; Tn(I) = troponin (I);

References

Araszkiewicz, A., Grygier, M., Pyda, M., Rajewska, J., Michalak, M., Lesiak, M., et al. (2014). Postconditioning reduces enzymatic infarct size and improves microvascular reperfusion in patients with ST-segment elevation myocardial infarction. Cardiology, 129, 250-257. https://doi.org/10.1159/000367965

Bodi, V., Ruiz-Nodar, J.M., Feliu, E., Minana, G., Nunez, J., Husser, O., et al. (2014). Effect of ischemic postconditioning on microvascular obstruction in reperfused myocardial infarction. Results of a randomized study in patients and of an experimental model in swine. International Journal of Cardiology, 175, 138-146. https://doi.org/10.1016/j.ijcard.2014.05.003

Bøtker, H.E., Kharbanda, R., Schmidt, M.R., Bottcher, M., Kaltoft, A.K., Terkelsen, C.J., et al. (2010). Remote ischaemic conditioning before hospital admission, as a complement to angioplasty, and effect on myocardial salvage in patients with acute myocardial infarction: a randomised trial. The Lancet, 375, 727-734. https://doi.org/10.1016/S0140-6736(09)62001-8

Cao, B., Zhang, C., Wang, H., Xia, M., & Yang, X. (2018). Renoprotective effect of remote ischemic postconditioning in patients with ST-elevation myocardial infarction undergoing primary percutaneous coronary intervention. Therapeutics and Clinical Risk Management, 14, 369-375. https://doi.org/10.2147/TCRM.S158768

Crimi, G., Pica, S., Raineri, C., Bramucci, E., De Ferrari, G.M., Klersy, C., et al. (2013). Remote ischemic post-conditioning of the lower limb during primary percutaneous coronary intervention safely reduces enzymatic infarct size in anterior myocardial infarction: a randomized controlled trial. Journal of the American College of Cardiology Cardiovascular Intervention, 6, 1055-1063. https://doi.org/10.1016/j.jcin.2013.05.011

Dong, M., Mu, N., Guo, F., Zhang, C., Ren, F., Li, J., et al. (2014). The beneficial effects of postconditioning on no-reflow phenomenon after percutaneous coronary intervention in

patients with ST-elevation acute myocardial infarction. Journal of Thrombosis and Thrombolysis, 38, 208-214. https://doi.org/10.1007/s11239-013-1010-0

Dwyer, N.B., Mikami, Y., Hilland, D., Aljizeeri, A., Friedrich, M.G., Traboulsi, M., et al. (2013). No cardioprotective benefit of ischemic postconditioning in patients with ST-segment elevation myocardial infarction. Journal of Interventional Cardiology, 26, 482-490. https://doi.org/ 10.1111/joic.12064

Eitel, I., Stiermaier, T., Rommel, K.P., Fuernau, G., Sandri, M., Mangner, N., et al. (2015). Cardioprotection by combined intrahospital remote ischaemic perconditioning and postconditioning in ST-elevation myocardial infarction: the randomized LIPSIA CONDITIONING trial. European Heart Journal, 36, 3049-3057. https://doi.org/10.1093/eurheartj/ehv463

Elbadawi, A., Awad, O., Raymond, R., Badran, H., Mostafa, A.E., & Saad, M. (2017). Impact of remote ischemic postconditioning during primary percutaneous coronary intervention on left ventricular remodeling after anterior wall ST-segment elevation myocardial infarction: A single-center experience. The International Journal of Angiology, 26, 241-248. https://doi.org/10.1055/s-0037-1601870

Elzbieciak, M., Wita, K., Grabka, M., Chmurawa, J., Doruchowska, A., Turski, M., et al. (2013). Effect of postconditioning on infarction size, adverse left ventricular remodeling, and improvement in left ventricular systolic function in patients with first anterior STsegment elevation myocardial infarction. Polish Archives of Internal Medicine, 123, 268-276. https://doi.org/10.20452/pamw.1766

Engstrøm, T., Kelbaek, H., Helqvist, S., Hofsten, D.E., Klovgaard, L., Clemmensen, P., et al. (2017). Effect of ischemic postconditioning during primary percutaneous coronary intervention for patients with ST-segment elevation myocardial infarction: A Randomized Clinical Trial. The Journal of the American Medical Association Cardiology, 2, 490-497. https://doi.org/10.1001/jamacardio.2017.0022

Freixa, X., Bellera, N., Ortiz-Perez, J.T., Jimenez, M., Pare, C., Bosch, X., et al. (2012). Ischaemic postconditioning revisited: lack of effects on infarct size following primary percutaneous coronary intervention. European Heart Journal, 33, 103-112. https://doi.org/ 10.1093/eurheartj/ehr297

Garcia, S., Henry, T.D., Wang, Y.L., Chavez, I.J., Pedersen, W.R., Lesser, J.R., et al. (2011). Long-term follow-up of patients undergoing postconditioning during ST-elevation myocardial

infarction. Journal of Cardiovascular Translational Research 4, 92-98. https://doi.org/10.1007/s12265-010-9252-0

Gaspar, A., Lourenco, A.P., Pereira, M.A., Azevedo, P., Roncon-Albuquerque, R., Jr., Marques, J., et al. (2018). Randomized controlled trial of remote ischaemic conditioning in ST-elevation myocardial infarction as adjuvant to primary angioplasty (RIC-STEMI). Basic Research in Cardiology, 113, 14. https://doi.org/10.1007/s00395-018-0672-3

Ghaffari, S., Pourafkari, L., Manzouri, S., & Nader, N.D. (2018). Effect of remote ischemic postconditioning during thrombolysis in STEMI. Herz, 43, 161-168. https://doi.org/10.1007/s00059-017-4550-9

Hahn, J.Y., Song, Y.B., Kim, E.K., Yu, C.W., Bae, J.W., Chung, W.Y., et al. (2013). Ischemic postconditioning during primary percutaneous coronary intervention: The POST Randomized Trial. Circulation, 128, 1889-1896. https://doi.org/10.1161/CIRCULATIONAHA.113.001690.

Kim, E.K., Hahn, J.Y., Song, Y.B., Lee, S.C., Choi, J.H., Choi, S.H., et al. (2015). Effect of ischemic postconditioning on myocardial salvage in patients undergoing primary percutaneous coronary intervention for ST-segment elevation myocardial infarction: cardiac magnetic resonance substudy of the POST randomized trial. International Journal of Cardiovascular Imaging 31, 629-637. https://doi.org/10.1007/s10554-015-0589-y

Ladejobi, A., Wayne, M., Martin-Gill, C., Guyette, F.X., Althouse, A.D., Sharbaugh, M.S., et al. (2017). Association of remote ischemic peri-conditioning with reduced incidence of clinical heart failure after primary percutaneous coronary intervention. Cardiovascular Revascularization Medicine, 18, 105-109. https://doi.org/10.1016/j.carrev.2016.12.004

Laskey, W.K., Yoon, S., Calzada, N., & Ricciardi, M.J. (2008). Concordant improvements in coronary flow reserve and ST-segment resolution during percutaneous coronary intervention for acute myocardial infarction: a benefit of postconditioning. Catheter Cardiovascular Intervention, 72, 212-220. https://doi.org/10.1002/ccd.21583

Limalanathan, S., Andersen, G.O., Klow, N.E., Abdelnoor, M., Hoffmann, P., & Eritsland, J. (2014). Effect of ischemic postconditioning on infarct size in patients with ST-elevation myocardial infarction treated by primary PCI results of the POSTEMI (POstconditioning in ST-Elevation Myocardial Infarction) randomized trial. J Am Heart Assoc, 3, e000679. https://doi.org/10.1161/JAHA.113.000679

Liu, S.H., Huo, Y.E., Yin, B.Y., Li, X.H., & Wang, Y.F. (2013). Ischemic postconditioning may increase serum fetuin-A level in patients with acute ST-segment elevation myocardial infarction undergoing percutaneous intervention. Clinical Laboratory, 59, 59-64. https://doi.org/10.7754/Clin.Lab.2012.120222

Liu, T.K., Mishra, A.K., & Ding, F.X. (2011). Protective effect of ischemia postconditioning on reperfusion injury in patients with ST-segment elevation acute myocardial infarction. Chinese Journal of Cardiology, 39, 35-39. https://doi.org/10.3760/cma.j.issn.0253-3758.2011.01.007

Liu, Z., Zhao, L., Hong, D., & Gao, J. (2016). Remote ischaemic preconditioning reduces myocardial ischaemic reperfusion injury in patients with ST-elevation myocardial infarction undergoing primary percutaneous coronary intervention. Acta cardiologica, 71, 596-603. https://doi.org/10.2143/AC.71.5.3167504

Lonborg, J., Kelbaek, H., Vejlstrup, N., Jorgensen, E., Helqvist, S., Saunamaki, K., et al. (2010). Cardioprotective effects of ischemic postconditioning in patients treated with primary percutaneous coronary intervention, evaluated by magnetic resonance. Circulation Cardiovascular Intervention, 3, 34-41. https://doi.org/10.1161/CIRCINTERVENTIONS.109.905521

Luz, A., Santos, M., Rodrigues, P., Sousa, M.J., Anjo, D., Silveira, I., et al. (2015). Preinfarction angina: clinical significance and relationship with total ischemic time in patients with ST-elevation myocardial infarction. Coronary Artery Disease, 26, 22-29.

Ma, X.J., Zhang, X.H., Li, C.M., & Luo, M. (2006). Effect of postconditioning on coronary blood flow velocity and endothelial function in patients with acute myocardial infarction. Scandinavian Cardiovascular Journal, 40, 327-333. https://doi.org/10.1080/14017430601047864

Mewton, N., Thibault, H., Roubille, F., Lairez, O., Rioufol, G., Sportouch, C., et al. (2013). Postconditioning attenuates no-reflow in STEMI patients. Basic Research in Cardiology, 108, 383. https://doi.org/10.1007/s00395-013-0383-8.

Munk, K., Andersen, N.H., Schmidt, M.R., Nielsen, S.S., Terkelsen, C.J., Sloth, E., et al. (2010). Remote ischemic conditioning in patients with myocardial infarction treated with primary angioplasty: Impact on left ventricular function assessed by comprehensive echocardiography and gated single-photon emission CT. Circulation Cardiovascular Imaging, 3, 656-662. https://doi.org/10.1161/CIRCIMAGING.110.957340

Prunier, F., Angoulvant, D., Saint Etienne, C., Vermes, E., Gilard, M., Piot, C., et al. (2014). The RIPOST-MI study, assessing remote ischemic perconditioning alone or in combination with local ischemic postconditioning in ST-segment elevation myocardial infarction. Basic Research in Cardiology, 109, 400. https://doi.org/10.1007/s00395-013-0400-y

Rentoukas, I., Giannopoulos, G., Kaoukis, A., Kossyvakis, C., Raisakis, K., Driva, M., et al. (2010). Cardioprotective role of remote ischemic periconditioning in primary percutaneous coronary intervention: enhancement by opioid action. Journal of the American College of Cardiology: Cardiovascular Intervention, 3, 49-55. https://doi.org/10.1016/j.jcin.2009.10.015

Sörensson, P., Saleh, N., Bouvier, F., Bohm, F., Settergren, M., Caidahl, K., et al. (2010). Effect of postconditioning on infarct size in patients with ST elevation myocardial infarction. Heart, 96, 1710-1715. https://doi.org/10.1136/hrt.2010.199430

Staat, P., Rioufol, G., Piot, C., Cottin, Y., Cung, T.T., L'Huillier, I., et al. (2005). Postconditioning the human heart. Circulation, 112, 2143-2148. https://doi.org/10.1161/CIRCULATIONAHA.105.558122.

Tarantini, G., Favaretto, E., Marra, M.P., Frigo, A.C., Napodano, M., Cacciavillani, L., et al. (2012). Postconditioning during coronary angioplasty in acute myocardial infarction: the POST-AMI trial. International Journal of Cardiology, 162, 33-38. https://doi.org/10.1016/j.ijcard.2012.03.136

Thibault, H., Piot, C., Staat, P., Bontemps, L., Sportouch, C., Rioufol, G., et al. (2008). Long-term benefit of postconditioning. Circulation, 117, 1037-1044. https://doi.org/10.1161/CIRCULATIONAHA.107.729780.

Thuny, F., Lairez, O., Roubille, F., Mewton, N., Rioufol, G., Sportouch, C., et al. (2012). Post-conditioning reduces infarct size and edema in patients with ST-segment elevation myocardial infarction. Journal of the American College of Cardiology, 59, 2175-2181. https://doi.org/10.1016/j.jacc.2012.03.026.

Traverse, J.H., Swingen, C., Henry, T.D., Fox, J., Wang, Y., Chavez, I., et al. (2019). NHLBI-sponsored trial of postconditioning during primary percutaneous coronary intervention for ST-elevation myocardial infarction. Circulation Research, 124, 769–778. https://doi.org/10.1161/CIRCRESAHA.118.314060

Ugata, Y., Nakamura, T., Taniguchi, Y., Ako, J., & Momomura, S. (2012). Effect of postconditioning in patients with ST-elevation acute myocardial infarction. Cardiovascular Intervention and Therapeutics 27, 14-18. https://doi.org/10.1007/s12928-011-0077-9

White, S.K., Froehlich, G.M., Sado, D.M., Maestrini, V., Fontana, M., Treibel, T.A., et al. (2015). Remote ischemic conditioning reduces myocardial infarct size and edema in patients with ST-segment elevation myocardial infarction. Journal of the American College of Cardiology: Cardiovascular Intervention, 8, 178-188. https://doi.org/10.1016/j.jcin.2014.05.015

Xue, F., Yang, X., Zhang, B., Zhao, C., Song, J., Jiang, T., et al. (2010). Postconditioning the human heart in percutaneous coronary intervention. Clinical Cardiology, 33, 439-444. https://doi.org/10.1002/clc.20796

Yamanaka, T., Kawai, Y., Miyoshi, T., Mima, T., Takagaki, K., Tsukuda, S., et al. (2015). Remote ischemic preconditioning reduces contrast-induced acute kidney injury in patients with ST-elevation myocardial infarction: A randomized controlled trial. International Journal of Cardiology, 178C, 136-141. https://doi.org/10.1016/j.ijcard.2014.10.135

Yang, X.C., Liu, Y., Wang, L.F., Cui, L., Wang, T., Ge, Y.G., et al. (2007). Reduction in myocardial infarct size by postconditioning in patients after percutaneous coronary intervention. Journal of Invasive Cardiology, 19, 424-430.

Yellon, D.M., Ackbarkhan, A.K., Balgobin, V., Bulluck, H., Deelchand, A., Dhuny, M.R., et al. (2015). Remote ischemic conditioning reduces myocardial infarct size in STEMI patients treated by thrombolysis. Journal of the American College of Cardiology, 65, 2764-2765. https://doi.org/10.1016/j.jacc.2015.02.082

Yetgin, T., Magro, M., Manintveld, O.C., Nauta, S.T., Cheng, J.M., den Uil, C.A., et al. (2014). Impact of multiple balloon inflations during primary percutaneous coronary intervention on infarct size and long-term clinical outcomes in ST-segment elevation myocardial infarction: real-world postconditioning. Basic Research in Cardiology, 109, 403. https://doi.org/10.1007/s00395-014-0403-3

Zhao, W.S., Xu, L., Wang, L.F., Zhang, L., Zhang, Z.Y., Liu, Y., et al. (2009). A 60-s postconditioning protocol by percutaneous coronary intervention inhibits myocardial apoptosis in patients with acute myocardial infarction. Apoptosis, 14, 1204-1211. https://doi.org/10.1007/s10495-009-0387-x

Zhou, F.Z., Song, W., Yin, L.H., Song, Z.F., Yang, S., Yang, F.B., et al. (2017). Effects of remote ischemic preconditioning on myocardial injury and endothelial function and prognosis after percutaneous coronary intervention in patients with acute coronary syndrome. European Review for Medical and Pharmacological Sciences, 21, 4642-4648.
