# Supplementary material for: Co‐morbidities and co‐medications as confounders of cardioprotection—Does it matter in the clinical setting?
Source: Br J Pharmacol. 2020 Jan 3;177(23):5252–69. doi: 10.1111/bph.14839 (PMC7680006; doi:10.1111/bph.14839)
Supplement: Supplementary file 2 — Figure S2: Forest plot of clinical studies on pharmacological conditioning in patients with acute myocardial infarction and with biomarker release or imaging techniques to estimate infarct size as end‐point. The zero represents the mean value, and the gray bars represent the standard error of the mean for the placebo group. Closed squares represent significantly reduced infarct size (x ~ ± SEM), open squares represent non‐significant changes. The star on the midline is used when no information was given to the standard error of the mean. ANP = atrial natriuretic peptide; CK(‐MB) = creatine kinase (muscle‐brain); drug = pharmacological conditioning group; Fx06 = peptide derived from the neo‐N‐terminus of fibrin; G‐CSF = granulocyte‐colony stimulating factor; GIK = glucose‐insulin‐potassium; (hs)Tn(I/T) = (high sensitive) troponin (I/T); IGF1 = insulin‐like growth factor 1; LeukArrest = rovelizumab; n.s. = not significant; MRI = magnetic resonance imaging; MTP‐131 = cardiolipin‐targeting peptide; PLA = placebo group; SNP = sodium nitroprusside; SPECT = single‐photon emission computed tomography; TRO40303 = 3,5‐seco‐4‐nor‐cholestan‐5‐one oxime‐3‐ol; * = dual anti platelet therapy [file BPH-177-5252-s002.pdf]

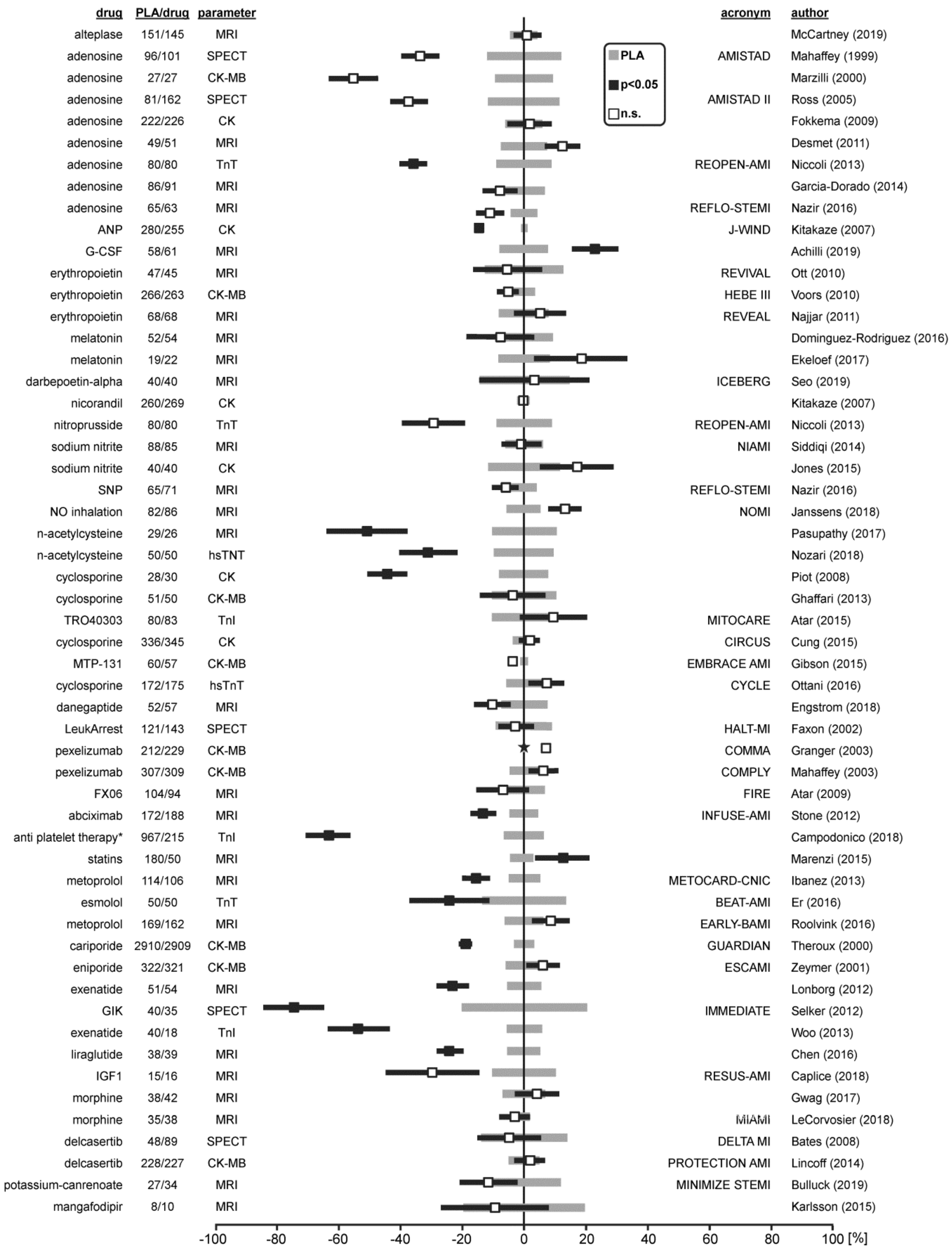

Supplemental Figure 2: Forest plot of clinical studies on pharmacological conditioning in patients with acute myocardial infarction and with biomarker release or imaging techniques to estimate infarct size as end-point. The zero represents the mean value, and the gray bars represent the standard error of the mean for the placebo group. Closed squares represent significantly reduced infarct size ($\tilde{x}$ ±SEM), open squares represent non-significant changes.

The star on the midline is used when no information was given to the standard error of the mean. ANP = atrial natriuretic peptide; CK(-MB) = creatine kinase (muscle-brain); drug = pharmacological conditioning group; Fx06 = peptide derived from the neo-N-terminus of fibrin; G-CSF = granulocyte-colony stimulating factor; GIK = glucose-insulin-potassium; (hs)Tn(I/T) = (high sensitive) troponin (I/T); IGF1 = insulin-like growth factor 1; LeukArrest = rovelizumab; n.s. = not significant; MRI = magnetic resonance imaging; MTP-131 = cardiolipin-targeting peptide; PLA = placebo group; SNP = sodium nitroprusside; SPECT = single-photon emission computed tomography; TRO40303 = 3,5-seco-4-nor-cholestan-5-one oxime-3-ol; * = dual anti platelet therapy

References

Achilli, F., Pontone, G., Bassetti, B., Squadroni, L., Campodonico, J., Corrada, E., et al. (2019). G-CSF for Extensive STEMI: Results from the STEM-AMI OUTCOME CMR Substudy. Circulation Research, https://doi.org/10.1161/CIRCRESAHA.118.314617

Atar, D., Arheden, H., Berdeaux, A., Bonnet, J.L., Carlsson, M., Clemmensen, P., et al. (2015). Effect of intravenous TRO40303 as an adjunct to primary percutaneous coronary intervention for acute ST-elevation myocardial infarction: MITOCARE study results. European Heart Journal, 36, 112-119. https://doi.org/10.1093/eurheartj/ehu331.

Atar, D., Petzelbauer, P., Schwitter, J., Huber, K., Rensing, B., Kasprzak, J.D., et al. (2009). Effect of intravenous FX06 as an adjunct to primary percutaneous coronary intervention for acute ST-segment elevation myocardial infarction results of the F.I.R.E. (Efficacy of FX06 in the Prevention of Myocardial Reperfusion Injury) trial. Journal of the American College of Cardiology, 53, 720-729. https://doi.org/10.1016/j.jacc.2008.12.017

Bates, E., Bode, C., Costa, M., Gibson, C.M., Granger, C., Green, C., et al. (2008). Intracoronary KAI-9803 as an adjunct to primary percutaneous coronary intervention for acute ST-segment elevation myocardial infarction. Circulation, 117, 886-896. https://doi.org/10.1161/CIRCULATIONAHA.107.759167

Bulluck, H., Froehlich, G.M., Nicholas, J.M., Mohdnazri, S., Gamma, R., Davies, J., et al. (2019). Mineralocorticoid receptor antagonist pre-treatment and early post-treatment to

minimize reperfusion injury after ST-elevation myocardial infarction: The MINIMIZE STEMI trial. American Heart Journal, 211, 60-67. https://doi.org/10.1016/j.ahj.2019.02.005

Campodonico, J., Cosentino, N., Milazzo, V., Rubino, M., De Metrio, M., Marana, I., et al. (2018). Impact of chronic antiplatelet therapy on infarct size and bleeding in patients with acute myocardial infarction. Journal of Cardiovascular Pharmacology and Therapeutics, 23, 407-413. https://doi.org/10.1177/1074248418769636

Caplice, N.M., DeVoe, M.C., Choi, J., Dahly, D., Murphy, T., Spitzer, E., et al. (2018). Randomized placebo controlled trial evaluating the safety and efficacy of single low-dose intracoronary insulin-like growth factor following percutaneous coronary intervention in acute myocardial infarction (RESUS-AMI). American Heart Journal, 200, 110-117. https://doi.org/10.1016/j.ahj.2018.03.018

Chen, W.R., Chen, Y.D., Tian, F., Yang, N., Cheng, L.Q., Hu, S.Y., et al. (2016). Effects of liraglutide on reperfusion injury in patients with ST-segment-elevation myocardial infarction. Circulation Cardiovascular Imaging, 9, e005146. https://doi.org/10.1161/CIRCIMAGING.116.005146

Cung, T.T., Morel, O., Cayla, G., Rioufol, G., Garcia-Dorado, D., Angoulvant, D., et al. (2015). Cyclosporine before PCI in patients with acute myocardial infarction. The New England Journal of Medicine, 373, 1021-1103. https://doi.org/10.1056/NEJMoa1505489

Desmet, W., Bogaert, J., Dubois, C., Sinnaeve, P., Adriaenssens, T., Pappas, C., et al. (2011). High-dose intracoronary adenosine for myocardial salvage in patients with acute ST-segment elevation myocardial infarction. European Heart Journal, 32, 867-877. https://doi.org/10.1093/eurheartj/ehq492.

Dominguez-Rodriguez, A., Abreu-Gonzalez, P., de la Torre-Hernandez, J.M., Gonzalez-Gonzalez, J., Garcia-Camarero, T., Consuegra-Sanchez, L., et al. (2017). Effect of intravenous and intracoronary melatonin as an adjunct to primary percutaneous coronary intervention for acute ST-elevation myocardial infarction: Results of the melatonin adjunct in the acute myocardial infarction treated with angioplasty trial. Journal of Pineal Research, 62, e12374. https://doi.org/10.1111/jpi.12374

Ekeloef, S., Halladin, N., Fonnes, S., Jensen, S.E., Zaremba, T., Rosenberg, J., et al. (2017). Effect of intracoronary and intravenous melatonin on myocardial salvage index in patients

with ST-elevation myocardial infarction: a randomized placebo controlled trial. Journal of Cardiovascular Translational Research, 10, 470-479. https://doi.org/10.1007/s12265-017-9768-7

Engstrøm, T., Nepper-Christensen, L., Helqvist, S., Klovgaard, L., Holmvang, L., Jørgensen, E., et al. (2018). Danegaptide for primary percutaneous coronary intervention in acute myocardial infarction patients: a phase 2 randomised clinical trial. Heart, 104, 1593-1599. https://doi.org/10.1136/heartjnl-2017-312774

Er, F., Dahlem, K.M., Nia, A.M., Erdmann, E., Waltenberger, J., Hellmich, M., et al. (2016). Randomized control of sympathetic drive with continuous esmolol in patients with acute ST-segment elevation myocardial infarction. The BEtA-Blocker Therapy in acute myocardial infarction (BEAT-AMI) Trial. Journal of the American College of Cardiology: Cardiovascular Intervention, 9, 231-240. https://doi.org/10.1016/j.jcin.2015.10.035

Faxon, D.P., Gibbons, R.J., Chronos, N.A., Gurbel, P.A., & Sheehan, F. (2002). The effect of blockade of the CD11/CD18 integrin receptor on infarct size in patients with acute myocardial infarction treated with direct angioplasty: the results of the HALT-MI study. Journal of the American College of Cardiology, 40, 1199-1204. https://doi.org/10.1016/S0735-1097(02)02136-8

Fokkema, M.L., Vlaar, P.J., Vogelzang, M., Gu, Y.L., Kampinga, M.A., de Smet, B.J., et al. (2009). Effect of high-dose intracoronary adenosine administration during primary percutaneous coronary intervention in acute myocardial infarction: a randomized controlled trial. Circulation Cardiovascular Intervention, 2, 323-329. https://doi.org/doi: 10.1161/CIRCINTERVENTIONS.109.858977.109.858977.

Garcia-Dorado, D., Garcia-Del-Blanco, B., Otaegui, I., Rodriguez-Palomares, J., Pineda, V., Gimeno, F., et al. (2014). Intracoronary injection of adenosine before reperfusion in patients with ST-segment elevation myocardial infarction: A randomized controlled clinical trial. International Journal of Cardiology, 177, 935-941. https://doi.org/10.1016/j.ijcard.2014.09.203.

Ghaffari, S., Kazemi, B., Toluey, M., & Sepehrvand, N. (2013). The effect of prethrombolytic cyclosporine-A injection on clinical outcome of acute anterior ST-elevation myocardial infarction. Cardiovascular Therapeutics, 31, e34-39. https://doi.org/10.1111/1755-5922.12010

Gibson, C.M., Giugliano, R.P., Kloner, R.A., Bode, C., Tendera, M., Janosi, A., et al. (2015). EMBRACE STEMI study: a Phase 2a trial to evaluate the safety, tolerability, and efficacy of intravenous MTP-131 on reperfusion injury in patients undergoing primary percutaneous coronary intervention. European Heart Journal, 37, 1296-1303. https://doi.org/10.1093/eurheartj/ehv597

Granger, C.B., Mahaffey, K.W., Weaver, W.D., Theroux, P., Hochman, J.S., Filloo, T.G., et al. (2003). Effect of pexelizumab, an anti-C5 complement antibody, as adjunctive therapy toprimary percutaneous coronary intervention in acute myocardial infarction. The COMPlement inhibition in myocardial infarction treated with angioplasty(COMMA) trial. Circulation, 108, 1184-1190. https://doi.org/10.1161/01.CIR.0000087447.12918.85

Gwag, H.B., Kim, E.K., Park, T.K., Lee, J.M., Yang, J.H., Song, Y.B., et al. (2017). Cardioprotective effects of intracoronary morphine in ST-segment elevation myocardial infarction patients undergoing primary percutaneous coronary intervention: A prospective, randomized trial. J Am Heart Assoc, 6, e005426. https://doi.org/10.1161/JAHA.116.005426

Ibanez, B., Macaya, C., Sanchez-Brunete, V., Pizarro, G., Fernandez-Friera, L., Mateos, A., et al. (2013). Effect of early metoprolol on infarct size in ST-segment-elevation myocardial infarction patients undergoing primary percutaneous coronary intervention: The effect of metoprolol in cardioprotection during an acute myocardial infarction (METOCARD-CNIC) trial. Circulation, 128, 1495-1503. https://doi.org/10.1161/CIRCULATIONAHA.113.003653.

Janssens, S.P., Bogaert, J., Zalewski, J., Toth, A., Adriaenssens, T., Belmans, A., et al. (2018). Nitric oxide for inhalation in ST-elevation myocardial infarction (NOMI): a multicentre, double-blind, randomized controlled trial. European Heart Journal, 39, 2717-2725. https://doi.org/10.1093/eurheartj/ehy232

Jones, D.A., Pellaton, C., Velmurugan, S., Andiapen, M., Antoniou, S., van Eijl, S., et al. (2015). Randomized phase 2 trial of intra-coronary nitrite during acute myocardial infarction. Circulation Research, 116, 437-447. https://doi.org/10.1161/CIRCRESAHA.116.305082.

Karlsson, J.E., El-Saadi, W., Ali, M., Puskar, W., Skogvard, P., Engvall, J.E., et al. (2015). Mangafodipir as a cardioprotective adjunct to reperfusion therapy: a feasibility study in

patients with ST-segment elevation myocardial infarction. European Heart Journal of Cardiovascular Pharmacotherapy, 1, 39-45. https://doi.org/10.1093/ehjcvp/pvu021

Kitakaze, M., Asakura, M., Kim, J., Shintani, Y., Asanuma, H., Hamasaki, T., et al. (2007). Human atrial natriuretic peptide and nicorandil as adjuncts to reperfusion treatment for acute myocardial infarction (J-WIND): two randomised trials. The Lancet, 370, 1483-1493. https://doi.org/10.1016/S0140-6736(07)61634-1.

Le Corvoisier, P., Gallet, R., Lesault, P.F., Audureau, E., Paul, M., Ternacle, J., et al. (2018). Intra-coronary morphine versus placebo in the treatment of acute ST-segment elevation myocardial infarction: the MIAMI randomized controlled trial. British Medical Journal Cardiovascular Disorders, 18, 193. https://doi.org/10.1186/s12872-018-0936-8

Lincoff, A.M., Roe, M., Aylward, P., Galla, J., Rynkiewicz, A., Guetta, V., et al. (2014). Inhibition of delta-protein kinase C by delcasertib as an adjunct to primary percutaneous coronary intervention for acute anterior ST-segment elevation myocardial infarction: results of the PROTECTION AMI Randomized Controlled Trial. European Heart Journal, 35, 2516-2523. https://doi.org/10.1093/eurheartj/ehu177.

Lonborg, J., Vejlstrup, N., Kelbaek, H., Bøtker, H.E., Kim, W.Y., Mathiasen, A.B., et al. (2012). Exenatide reduces reperfusion injury in patients with ST-segment elevation myocardial infarction. European Heart Journal, 33, 1491-1499. https://doi.org/10.1093/eurheartj/ehr309

Mahaffey, K.W., Granger, C.B., Nicolau, J.C., Ruzyllo, W., Weaver, W.D., Theroux, P., et al. (2003). Effect of pexelizumab, an anti-C5 complement antibody, as adjunctive therapy to fibrinolysis in acute myocardial infarction. The COMPlement inhibition in myocardial infarction treated with thromboLYtics (COMPLY) trial. Circulation, 108, 1176-1183. https://doi.org/10.1161/01.CIR.0000087404.53661.F8

Mahaffey, K.W., Puma, J.A., Barbagelata, A., DiCarli, M.F., Leesar, M.A., Browne, K.F., et al. (1999). Adenosine as an adjunct thrombolytic therapy for acute myocardial infarction. Journal of the American College of Cardiology, 34, 1711-1720.

Marenzi, G., Cosentino, N., Cortinovis, S., Milazzo, V., Rubino, M., Cabiati, A., et al. (2015). Myocardial infarct size in patients on long-term statin therapy undergoing primary

percutaneous coronary intervention for ST-elevation myocardial infarction. American Journal of Cardiology, 116, 1791-1797. https://doi.org/10.1016/j.amjcard.2015.09.016

Marzilli, M., Orsini, E., Marraccini, P., & Testa, R. (2000). Beneficial effects of intracoronary adenosine as an adjunct to primary angioplasty in acute myocardial infarction. Circulation, 101, 2154-2159. https://doi.org/10.1161/01.CIR.101.18.2154

McCartney, P.J., Eteiba, H., Maznyczka, A.M., McEntegart, M., Greenwood, J.P., Muir, D.F., et al. (2019). Effect of Low-Dose Intracoronary Alteplase During Primary Percutaneous Coronary Intervention on Microvascular Obstruction in Patients With Acute Myocardial Infarction: A Randomized Clinical Trial. Journal of the American Medical Association, 321, 56-68. https://doi.org/10.1001/jama.2018.19802

Najjar, S.S., Rao, S.V., Melloni, C., Raman, S.V., Povsic, T.J., Melton, L., et al. (2011). Intravenous erythropoietin in patients with ST-segment elevation myocardial infarction: REVEAL: a randomized controlled trial. Journal of the American Medical Association, 305, 1863-1872. https://doi.org/10.1001/jama.2011.592

Nazir, S.A., McCann, G.P., Greenwood, J.P., Kunadian, V., Khan, J.N., Mahmoud, I.Z., et al. (2016). Strategies to attenuate micro-vascular obstruction during P-PCI: the randomized reperfusion facilitated by local adjunctive therapy in ST-elevation myocardial infarction trial. European Heart Journal, 37, 1910-1919. https://doi.org/10.1093/eurheartj/ehw136

Niccoli, G., Rigattieri, S., De Vita, M.R., Valgimigli, M., Corvo, P., Fabbiocchi, F., et al. (2013). Open-label, randomized, placebo-controlled evaluation of intracoronary adenosine or nitroprusside after thrombus aspiration during primary percutaneous coronary intervention for the prevention of microvascular obstruction in acute myocardial infarction: The REOPEN-AMI Study. Journal of the American College of Cardiology Cardiovascular Intervention 6, 580-589.
https://doi.org/10.1016/j.jcin.2013.02.009

Nozari, Y., Eshraghi, A., Talasaz, A.H., Bahremand, M., Salamzadeh, J., Salarifar, M., et al. (2018). Protection from reperfusion injury with intracoronary N-acetylcysteine in patients with STEMI undergoing primary percutaneous coronary intervention in a cardiac tertiary center. American Journal of Cardiovascular Drugs : Drugs, Devices, and other Interventions, 18, 213-221. https://doi.org/10.1007/s40256-017-0258-8

Ott, I., Schulz, S., Mehilli, J., Fichtner, S., Hadamitzky, M., Hoppe, K., et al. (2010). Erythropoietin in patients with acute ST-segment elevation myocardial infarction undergoing primary percutaneous coronary intervention: a randomized, double-blind trial. Circulation Cardiovascular Intervention, 3, 408-413. https://doi.org/10.1161/CIRCINTERVENTIONS.109.904425

Ottani, F., Latini, R., Staszewsky, L., La Vecchia, L., Locuratolo, N., Sicuro, M., et al. (2016). Cyclosporine A in reperfused myocardial infarction. Themulticenter, contolled, open-label CYCLE Trial. Journal of the American College of Cardiology, 67, 365-374. https://doi.org/10.1016/j.jacc.2015.10.081

Pasupathy, S., Tavella, R., Grover, S., Raman, B., Procter, N.E.K., Du, Y.T., et al. (2017). Early use of N-acetylcysteine with nitrate therapy in patients undergoing primary percutaneous coronary intervention for ST-segment-elevation myocardial infarction reduces myocardial infarct size (the NACIAM Trial [N-acetylcysteine in Acute Myocardial Infarction]). Circulation, 136, 894-903. https://doi.org/10.1161/CIRCULATIONAHA.117.027575

Piot, C., Croisille, P., Staat, P., Thibault, H., Rioufol, G., Mewton, N., et al. (2008). Effect of cyclosporine on reperfusion injury in acute myocardial infarction. The New England Journal of Medicine, 359, 473-481. https://doi.org/10.1056/NEJMoa071142.

Roolvink, V., Ibanez, B., Ottervanger, J.P., Pizarro, G., van Royen, N., Mateos, A., et al. (2016). Early administration of intravenous beta blockers in patients with ST-elevation myocardial infarction before primary PCI. Journal of the American College of Cardiology, 67, 2705-2715. https://doi.org/10.1016/j.jacc.2016.03.522

Ross, A., Gibbons, R.J., Stone, G.W., Kloner, R.A., & Alexander, R.W. (2005). A randomized, double-blinded, placebo-controlled multicenter trial of adenosine as an adjunct to reperfusion in the treatment of acute myocardial infarction (AMISTAD II). Journal of the American College of Cardiology, 45, 1775-1780. https://doi.org/10.1016/j.jacc.2005.02.061.

Selker, H.P., Beshansky, J.R., Sheehan, P.R., Massaro, J.M., Griffith, J.L., D'Agostino, R.B., et al. (2012). Out-of-hospital administration of intravenous glucose-insulin-potassium in patients with suspected acute coronary syndromes: The IMMEDIATE Randomized Controlled Trial. Journal of the American Medical Association, 307, 1925-1933. https://doi.org/10.1001/jama.2012.426

Seo, W.W., Suh, J.W., Oh, I.Y., Yoon, C.H., Cho, Y.S., Youn, T.J., et al. (2019). Efficacy of IntraCoronary Erythropoietin delivery BEfore Reperfusion-Gauging infarct size in patients with acute ST-segment elevation myocardial infarction (ICEBERG). International Heart Journal, 60, 255-263. https://doi.org/10.1536/ihj.18-035

Siddiqi, N., Neil, C., Bruce, M., Maclennan, G., Cotton, S., Papadopoulou, S., et al. (2014). Intravenous sodium nitrite in acute ST-elevation myocardial infarction: a randomized controlled trial (NIAMI). European Heart Journal, 35, 1255-1262. https://doi.org/10.1093/eurheartj/ehu096.

Stone, G.W., Maehara, A., Witzenbichler, B., Godlewski, J., Parise, H., Dambrink, J.H., et al. (2012). Intracoronary abciximab and aspiration thrombectomy in patients with large anterior myocardial infarction: the INFUSE-AMI randomized trial. Journal of the American Medical Association, 307, 1817-1826. https://doi.org/10.1001/jama.2012.421

Theroux, P., Chaitman, B.R., Danchin, N., Erhardt, L., Meinertz, T., Schroeder, J.S., et al. (2000). Inhibition of the sodium-hydrogen exchanger with cariporide to prevent myocardial infarction in high-risk ischemic situations. Main results of the GUARDIAN trial. Circulation, 102, 3032-3038. https://doi.org/10.1161/01.CIR.102.25.3032

Voors, A.A., Belonje, A.M., Zijlstra, F., Hillege, H.L., Anker, S.D., Slart, R.H., et al. (2010). A single dose of erythropoietin in ST-elevation myocardial infarction. European Heart Journal, 31, 2593-2600. https://doi.org/10.1093/eurheartj/ehq304

Woo, J.S., Kim, W., Ha, S.J., Kim, J.B., Kim, S.J., Kim, W.S., et al. (2013). Cardioprotective effects of exenatide in patients with ST-segment-elevation myocardial infarction undergoing primary percutaneous coronary intervention: Results of exenatide myocardial protection in revascularization study. Arteriosclerotic Thrombosis Vascular Biology, 33, 2252-2260. https://doi.org/10.1161/ATVBAHA.113.301586.

Zeymer, U., Suryapranata, H., Monassier, J.P., Opolski, G., Davies, J., Rasmanis, G., et al. (2001). The Na+/H+ exchange inhibitor eniporide as an adjunct to early reperfusion therapy for acute myocardial infarction. Journal of the American College of Cardiology, 38, 1644-1650.
