# Supplementary material for: Co‐morbidities and co‐medications as confounders of cardioprotection—Does it matter in the clinical setting?
Source: Br J Pharmacol. 2020 Jan 3;177(23):5252–69. doi: 10.1111/bph.14839 (PMC7680006; doi:10.1111/bph.14839)
Supplement: Supplementary file 3 — Figure S3: Forest plot of clinical studies on ischaemic conditioning in patients undergoing cardiovascular surgery and with biomarker release as end‐point to estimate ischaemia/reperfusion injury. The zero represents the mean value, and the gray bars represent the standard error of the mean for the placebo group. Closed squares represent significantly reduced infarct size (x ~ ± SEM), open squares represent non‐significant changes. The star is used when no information was given to the standard error of the mean. CK‐MB = creatine kinase (muscle‐brain); (hs)Tn(I/T) = (high sensitive) troponin (I/T); ICU = propofol was given at the intensive care unit stay; IPC = local ischaemic preconditioning; n = no propofol anaesthesia; n.s. = not significant; PLA = placebo group; PoCo = local ischaemic postconditioning; RIC = remote ischaemic conditioning; RIC* = RIC in children undergoing cardiovascular surgery; RIC‐LT = repetitive long term RIC; y = yes, propofol anaesthesia;? = no information was given to the use of propofol [file BPH-177-5252-s003.pdf]

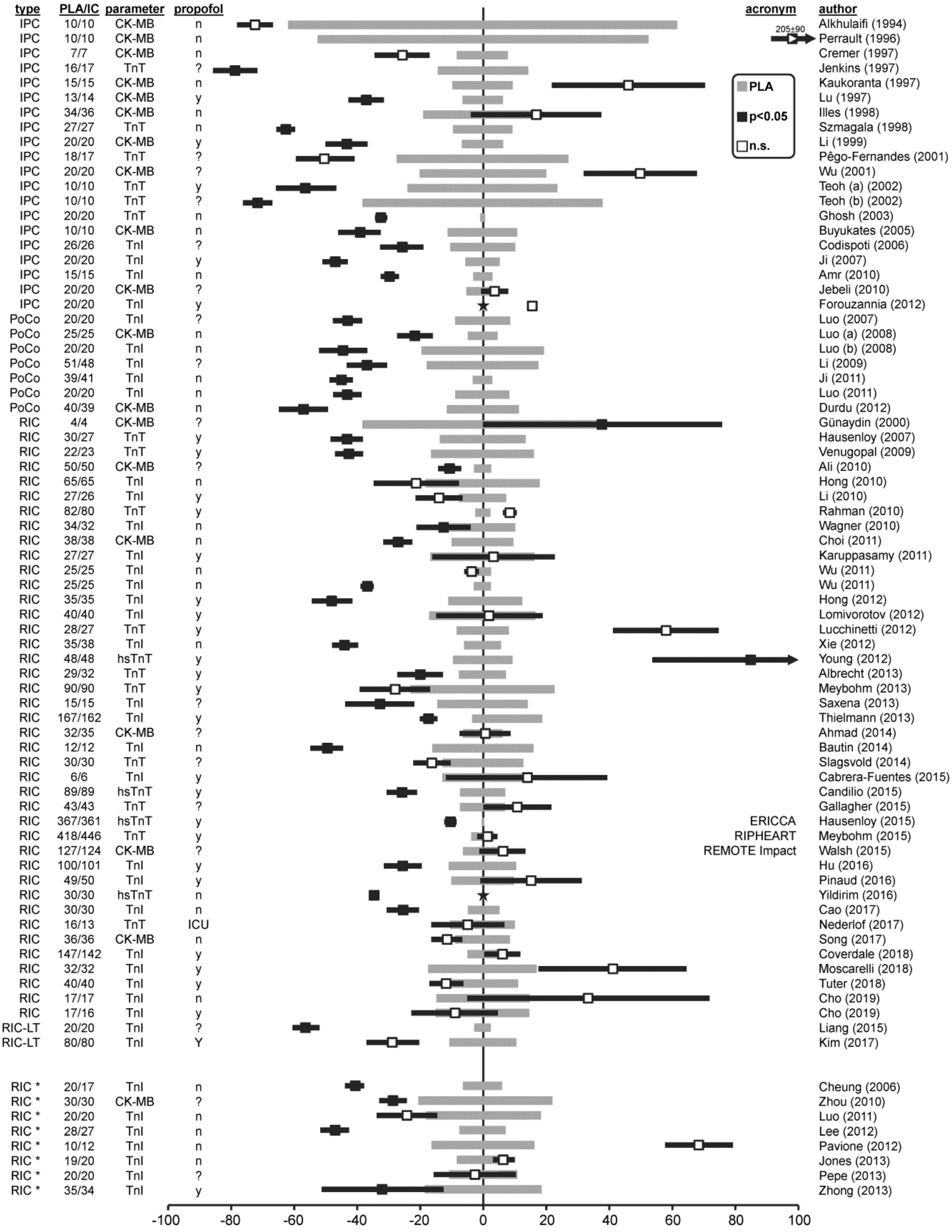

Supplemental Figure 3: Forest plot of clinical studies on ischaemic conditioning in patients undergoing cardiovascular surgery and with biomarker release as end-point to estimate ischaemia/reperfusion injury. The zero represents the mean value, and the gray bars represent the standard error of the mean for the placebo group. Closed squares represent significantly reduced infarct size ($\tilde{x}$ ±SEM), open squares represent non-significant changes. The star is used when no information was given to the standard error of the mean. CK-MB =

creatine kinase (muscle-brain); (hs)Tn(I/T) = (high sensitive) troponin (I/T); ICU = propofol was given at the intensive care unit stay; IPC = local ischaemic preconditioning; n= no propofol anaesthesia; n.s. = not significant; PLA = placebo group; PoCo = local ischaemic postconditioning; RIC = remote ischaemic conditioning; RIC* = RIC in children undergoing cardiovascular surgery; RIC-LT = repetitive long term RIC; y = yes, propofol anaesthesia; ? = no information was given to the use of propofol

References

Ahmad, A.M., Ali, G.S., & Tariq, W. (2014). Remote ischemic preconditioning is a safe adjuvant technique to myocardial protection but adds no clinical benefit after on-pump coronary artery bypass grafting. Heart Surgery Forum, 17, E220-E223. https://doi.org/10.1532/HSF98.2014391

Albrecht, M., Zitta, K., Bein, B., Wennemuth, G., Broch, O., Renner, J., et al. (2013). Remote ischemic preconditioning regulates HIF-1alpha levels, apoptosis and inflammation in heart tissue of cardiosurgical patients: a pilot experimental study. Basic Research in Cardiology, 108, 314. https://doi.org/ 10.1007/s00395-012-0314-0

Ali, N., Rizwi, F., Iqbal, A., & Rashid, A. (2010). Induced remote ischemic pre-conditioning on ischemia-reperfusion injury in patients undergoing coronary artery bypass. Journal of College of Physicians and Surgeons Pakistan, 20, 427-431. https://doi.org/ 07.2010/JCPSP.427431

Alkhulaifi, A.M., Yellon, D.M., & Pugsley, W.B. (1994). Preconditioning the human heart during aorto-coronary bypass surgery. European Journal of Cardio-Thoracic Surgery, 8, 270-275.

Amr, Y.M., & Yassin, I.M. (2010). Cardiac protection during on-pump coronary artery bypass grafting: ischemic versus isoflurane preconditioning. Seminars in Cardiothoracic and Vascular Anesthesia, 14, 205-211. https://doi.org/10.1177/1089253210376839

Bautin, A.E., Galagudza, M.M., Datsenko, S.V., Tashkhanov, D.M., Marichev, A.O., Bakanov, A., et al. (2014). Effects of remote ischemic preconditioning on perioperative period in elective aortic valve replacement. Anesteziol Reanimatol, 3, 11-17.

Buyukates, M., Kalaycioglu, S., Oz, E., & Soncul, H. (2005). Effects of ischemic preconditioning in human heart. Cardiovasc Surg, 20, 241-245. https://doi.org/10.1111/j.1540-8191.2005.200427.x

Cabrera-Fuentes, H.A., Niemann, B., Grieshaber, P., Wollbrueck, M., Gehron, J., Preissner, K.T., et al. (2015). RNase1 as a potential mediator of remote ischaemic preconditioning for cardioprotection. European Journal of Cardiothoracic Surgery 48, 732-737. https://doi.org/10.1093/ejcts/ezu519

Candilio, L., Malik, A., Ariti, C., Barnard, M., Di Salvo, C., Lawrence, D., et al. (2015). Effect of remote ischaemic preconditioning on clinical outcomes in patients undergoing cardiac bypass surgery: a randomised controlled clinical trial. Heart, 10, 185-192. https://doi.org/10.1136/heartjnl-2014-306178

Cao, Z., Shen, R., Zhang, X., Cheng, G., & Yan, Z. (2017). Effects of remote ischemic preconditioning on acute myocardial injury in patients undergoing valve replacement. Irish Journal of Medical Science, 186, 889-893. https://doi.org/10.1007/s11845-016-1521-8

Cheung, M.M., Kharbanda, R.K., Konstantinov, I.E., Shimizu, M., Frndova Meng, H., Li, J., et al. (2006). Randomized controlled trial of the effects of remote ischemic preconditioning on children undergoing cardiac surgery: first clinical application in humans. Journal of the American College of Cardiology, 47, 2277-2282. https://doi.org/10.1016/j.jacc.2006.01.066

Cho, Y.J., Nam, K., Kim, T.K., Choi, S.W., Kim, S.J., Hausenloy, D.J., et al. (2019). Sevoflurane, propofol and carvedilol block myocardial protection by limb remote ischemic preconditioning. International Journal of Cardiology, 20, 269. https://doi.org/10.3390/ijms20020269

Choi, Y.S., Shim, J.K., Kim, J.C., Kang, K.S., Seo, Y.H., Ahn, K.R., et al. (2011). Effect of remote ischemic preconditioning on renal dysfunction after complex valvular heart surgery: a randomized controlled trial. The Journal of Thoracic and Cardiovascular Surgery 142, 148-154. https://doi.org/10.1016/j.jtcvs.2010.11.018

Codispoti, M., Sundaramoorthi, T., Saad, R.A., Reid, A., Sinclair, C., & Mankad, P. (2006). Optimal myocardial protection strategy for coronary artery bypass grafting without cardioplegia: prospective randomised trial. Interactive Cardiovascular and Thoracic Surgery 5, 217-221. https://doi.org/10.1510/icvts.2005.116863

Coverdale, N.S., Hamilton, A., Petsikas, D., McClure, R.S., Malik, P., Milne, B., et al. (2018). Remote ischemic preconditioning in high-risk cardiovascular surgery patients: A Randomized-controlled Trial. Seminars in Thoracic and Cardiovascular Surgery, 30, 26-33. https://doi.org/10.1053/j.semtcvs.2017.09.001

Cremer, J., Steinhoff, G., Karck, M., Ahnsell, T., Brandt, M., Teebken, O.E., et al. (1997). Ischemic preconditioning prior to myocardial protection with cold blood cardioplegia in coronary surgery. European Journal of Cardio-Thoracic Surgery, 12, 753-758.

Durdu, S., Sirlak, M., Cetintas, D., Inan, M.B., Eryilmaz, S., Ozcinar, E., et al. (2012). The efficacies of modified mechanical post conditioning on myocardial protection for patients undergoing coronary artery bypass grafting. Journal Cardiothoracic Surgery, 7, 73. https://doi.org/10.1186/1749-8090-7-73

Forouzannia, S.K., Abdollahi, M.H., Mirhosseini, S.J., Hadadzadeh, M., Zarepur, R., Zarepur, E., et al. (2013). Adenosine preconditioning versus ischemic preconditioning in patients undergoing off-pump coronary artery bypass (OPCAB). The Journal of Tehran Heart Center, 8, 127-131.

Gallagher, E.J., & LeRoith, D. (2015). Obesity and diabetes: The increased risk of cancer and cancer-related mortality. Physiological Reviews, 95, 727-748. https://doi.org/10.1152/physrev.00030.2014

Ghosh, S., & Galinanes, M. (2003). Protection of the human heart with ischemic preconditioning during cardiac surgery: role of cardiopulmonary bypass. The Journal of Thoracic and Cardiovascular Surgery, 126, 133-142. https://doi.org/10.1016/S0022-5223(02)73293-5

Günaydin, B., Cakici, I., Soncul, H., Kalaycioglu, S., Cevik, C., Sancak, B., et al. (2000). Does remote organ ischaemia trigger cardiac preconditioning during coronary artery surgery? Pharmacological Research, 41, 493-496. https://doi.org/10.1006/phrs.1999.0611

Hausenloy, D.J., Candilio, L., Evans, R., Ariti, C., Jenkins, D.P., Kolvekar, S., et al. (2015). Remote ischemic preconditioning and outcomes of cardiac surgery. The New England Journal of Medicine, 373, 1408-1417. https://doi.org/10.1056/NEJMoa1413534

Hausenloy, D.J., Mwamure, P.K., Venugopal, V., Harris, J., Barnard, M., Grundy, E., et al. (2007). Effect of remote ischaemic preconditioning on myocardial injury in patients undergoing coronary artery bypass graft surgery: a randomized controlled trial. The Lancet, 370, 575-579. https://doi.org/ 10.1016/S0140-6736(07)61296-3

Hong, D.M., Jeon, Y., Lee, C.S., Kim, H.J., Lee, J.M., Bahk, J.H., et al. (2012). Effects of remote ischemic preconditioning with postconditioning in patients undergoing off-pump coronary artery bypass surgery. Circulation Journal, 76, 884-890. https://doi.org/10.1253/circj.CJ-11-1068

Hong, D.M., Mint, J.J., Kim, J.H., Sohn, I.S., Lim, T.W., Lim, Y.J., et al. (2010). The effect of remote ischaemic preconditioning on myocardial injury in patients undergoing off-pump coronary artery bypass graft surgery. Anaesthesia and Intensive Care 38, 924-929. https://doi.org/10.1177/0310057X1003800518

Hu, Q., Luo, W., Huang, L., Huang, R., Chen, R., & Gao, Y. (2016). Multiorgan protection of remote ischemic perconditioning in valve replacement surgery. The Journal of Surgical Research, 200, 13-20. https://doi.org/10.1016/j.jss.2015.06.053

Illes, R.W., & Swoyer, K.D. (1998). Prospective, randomized clinical study of ischemic preconditioning as an adjunct to intermittent cold blood cardioplegia. The Annals of Thoracic Surgery, 65, 748-753.

Jebeli, M., Esmaili, H.R., Mandegar, M.H., Rasouli, M.R., Eghtesadi-Araghi, P., Mohammadzadeh, R., et al. (2010). Evaluation of the effects of ischemic preconditioning with a short reperfusion phase on patients undergoing a coronary artery bypass graft. The Annals of Thoracic Surgery, 16, 248-252.

Jenkins, D.P., Pugsley, W.B., Alkhulaifi, A.M., Kemp, M., Hooper, J., & Yellon, D.M. (1997). Ischaemic preconditioning reduces troponin T release in patients undergoing coronary artery bypass surgery. Heart, 77, 314-318.

Ji, B., Liu, M., Liu, J., Wang, G., Feng, W., Lu, F., et al. (2007). Evaluation by cardiac troponin I: the effect of ischemic preconditioning as an adjunct to intermittent blood cardioplegia on coronary artery bypass grafting. Cardiovasc Surg, 22, 394-400. https://doi.org/10.1111/j.1540-8191.2007.00433.x

Ji, Q., Mei, Y., Wang, X., Feng, J., Wusha, D., Cai, J., et al. (2011). Effect of ischemic postconditioning in correction of tetralogy of Fallot. International Heart Journal 52, 312-317. https://doi.org/10.1536/ihj.52.312

Jones, B.O., Pepe, S., Sheeran, F.L., Donath, S., Hardy, P., Shekerdemian, L., et al. (2013). Remote ischemic preconditioning in cyanosed neonates undergoing cardiopulmonary bypass: A randomized controlled trial. The Journal of Thoracic and Cardiovascular Surgery, 146, 1334-1340. https://doi.org/10.1016/j.jtcvs.2013.01.003

Karuppasamy, P., Chaubey, S., Dew, T., Musto, R., Sherwood, R., Desai, J., et al. (2011). Remote intermittent ischemia before coronary artery bypass graft surgery: a strategy to reduce injury and inflammation? Basic Research in Cardiology, 106, 511-519. https://doi.org/10.1007/s00395-011-0185-9

Kaukoranta, P.K., Lepojarvi, M.P., Ylitalo, K.V., Kiviluoma, K.T., & Peuhkurinen, K.J. (1997). Normothermic retrograde blood cardioplegia with or without preceding ischemic preconditioning. The Annals of Thoracic Surgery, 63, 1268-1274.

Kim, T.K., Min, J.J., Cho, Y.J., Hausenloy, D.J., Ahn, H., Kim, K.H., et al. (2017). Effects of delayed remote ischemic preconditioning on peri-operative myocardial injury in patients undergoing cardiac surgery - A randomized controlled trial. International Journal of Cardiology, 227, 511-515. https://doi.org/10.1016/j.ijcard.2016.10.111

Lee, J.H., Park, Y.H., Byon, H.J., Kim, H.S., Kim, C.S., & Kim, J.T. (2012). Effect of remote ischaemic preconditioning on ischaemic-reperfusion injury in pulmonary hypertensive infants receiving ventricular septal defect repair. British Journal of Anaesthesiology, 108, 223-228. https://doi.org/ 10.1093/bja/aer388

Li, B., Chen, R., Huang, R., & Luo, W. (2009). Clinical benefit of cardiac ischemic postconditioning in corrections of tetralogy of Fallot. Interactive Cardiovascular and Thoracic Surgery, 8, 17-21. https://doi.org/10.1510/icvts.2008.189373

Li, G., Chen, S., Lu, E., & Li, Y. (1999). Ischemic preconditioning improves preservation with cold blood cardioplegia in valve replacement patients. European Journal of Cardio-Thoracic Surgery, 15, 653-657.

Li, L., Luo, W., Huang, L., Zhang, W., Gao, Y., Jiang, H., et al. (2010). Remote perconditioning reduces myocardial injury in adult valve replacement: a randomized controlled trial. The Journal of Surgical Research, 164, e21-e26. https://doi.org/10.1016/j.jss.2010.06.016

Liang, Y., Li, Y.P., He, F., Liu, X.Q., & Zhang, J.Y. (2015). Long-term, regular remote ischemic preconditioning improves endothelial function in patients with coronary heart disease. Brazilian Journal of Medical and Biological Research, 48, 568-576. https://doi.org/10.1590/1414-431X20144452

Lomivorotov, V.V., Shmyrev, V.A., Nepomnyaschih, V.A., Ponomarev, D.N., Knyazkova, L.G., Lomivorotov, V.N., et al. (2012). Remote ischaemic preconditioning does not protect the heart in patients undergoing coronary artery bypass grafting. Interactive Cardiovascular and Thoracic Surgery, 15, 18-22. https://doi.org/10.1093/icvts/ivs118

Lu, E.-X., Chen, S.-X., Yuan, M.-D., Hu, T.-H., Zhou, H.-C., Luo, W.-J., et al. (1997). Preconditioning improves myocardial preservation in patients undergoing open heart operations. The Annals of Thoracic Surgery, 64, 1320-1324.

Lucchinetti, E., Bestmann, L., Feng, J., Freidank, H., Clanachan, A.S., Finegan, B.A., et al. (2012). Remote ischemic preconditioning applied during isoflurane inhalation provides no benefit to the myocardium of patients undergoing on-pump coronary artery bypass graft surgery: Lack of synergy or evidence of antagonism in cardioprotection? Anesthesiology, 116, 296-310. https://doi.org/10.1097/ALN.0b013e318242349a

Luo, W., Li, B., Chen, R., Huang, R., & Lin, G. (2008a). Effect of ischemic postconditioning in adult valve replacement. European Journal of Cardio-Thoracic Surgery, 33, 203-208. https://doi.org/10.1016/j.ejcts.2007.11.010

Luo, W., Li, B., Lin, G., Chen, R., & Huang, R. (2008b). Does cardioplegia leave room for postconditioning in paediatric cardiac surgery? Cardiology in the Young 18, 282-287. https://doi.org/10.1017/S1047951108002072

Luo, W., Li, B., Lin, G., & Huang, R. (2007). Postconditioning in cardiac surgery for tetralogy of Fallot. Cardiovasc Surg, 133, 1373-1374. https://doi.org/10.1016/j.jtcvs.2007.01.028

Luo, W., Zhu, M., Huang, R., & Zhang, Y. (2011). A comparison of cardiac post-conditioning and remote pre-conditioning in paediatric cardiac surgery. Cardiology in the Young, 21, 266-270. https://doi.org/10.1017/S1047951110001915

Meybohm, P., Bein, B., Brosteanu, O., Cremer, J., Gruenewald, M., Stoppe, C., et al. (2015). A multicenter trial of remote ischemic preconditioning for heart surgery. The New England Journal of Medicine, 373, 1397-1407. https://doi.org/10.1056/NEJMoa1413579

Meybohm, P., Renner, J., Broch, O., Caliebe, D., Albrecht, M., Cremer, J., et al. (2013). Postoperative neurocognitive dysfunction in patients undergoing cardiac surgery after remote ischemic preconditioning: a double-blind randomized controlled pilot study. PLoS One, 8, e64743.

Moscarelli, M., Fiorentino, F., Suleiman, M.S., Emanueli, C., Reeves, B.C., Punjabi, P.P., et al. (2018). Remote ischaemic preconditioning in isolated aortic valve and coronary artery bypass surgery: a randomized trial. European Journal of Cardiothoracic Surgery https://doi.org/10.1093/ejcts/ezy404

Nederlof, R., Weber, N.C., Juffermans, N.P., de Mol, B.A., Hollmann, M.W., Preckel, B., et al. (2017). A randomized trial of remote ischemic preconditioning and control treatment for cardioprotection in sevoflurane-anesthetized CABG patients. BioMedical Central Journal Anesthesiology, 17, 51. https://doi.org/10.1186/s12871-017-0330-6

Pavione, M.A., Carmona, F., de, C.M., & Carlotti, A.P. (2012). Late remote ischemic preconditioning in children undergoing cardiopulmonary bypass: A randomized controlled trial. The Journal of Thoracic and Cardiovascular Surgery, 144, 178-183. https://doi.org/10.1016/j.jtcvs.2011.12.029

Pego-Fernandes, P.M., Jatene, F.B., Kwasnicka, K., Hueb, A.C., Moreira, L.F., Gentil, A.F., et al. (2000). Ischemic preconditioning in myocardial revascularization with intermittent aortic cross-clamping. Cardiovascular Surgery, 15, 333-338. https://doi.org/10.1111/j.1540-8191.2000.tb00467.x|

Pepe, S., Liaw, N.Y., Hepponstall, M., Sheeran, F.L., Yong, M.S., d'Udekem, Y., et al. (2013). Effect of remote ischemic preconditioning on phosphorylated protein signaling in children undergoing tetralogy of fallot repair: a randomized controlled trial. Journal of the American Heart Association, 2, e000095. https://doi.org/10.1161/JAHA.113.000095

Perrault, L.P., Menasche, P., Bel, A., de Chaumaray, T., Peynet, J., Mondry, A., et al. (1996). Ischemic preconditioning in cardiac surgery: a word of caution. The Journal of Thoracic and Cardiovascular Surgery, 112, 1378-1386.

Pinaud, F., Corbeau, J.J., Baufreton, C., Binuani, J.P., De Brux, J.L., Fouquet, O., et al. (2016). Remote ischemic preconditioning in aortic valve surgery: Results of a randomized controlled study. Journal of Cardiology, 67, 36-41. https://doi.org/10.1016/j.jjcc.2015.06.007

Rahman, I.A., Mascaro, J.G., Steeds, R.P., Frenneaux, M.P., Nightingale, P., Gosling, P., et al. (2010). Remote ischemic preconditioning in human coronary artery bypass surgery: from promise to disappointment? Circulation, 122, S53-S59. https://doi.org/10.1161/CIRCULATIONAHA.109.926667

Saxena, P., Aggarwal, S., Misso, N.L., Passage, J., Newman, M.A., Thompson, P.J., et al. (2013). Remote ischaemic preconditioning down-regulates kinin receptor expression in neutrophils of patients undergoing heart surgery. Interactive Cardiovascular and Thoracic Surgery, 17, 653-658. https://doi.org/10.1093/icvts/ivt279

Slagsvold, K.H., Rognmo, O., Hoydal, M., Wisloff, U., & Wahba, A. (2014). Remote ischemic preconditioning preserves mitochondrial function and influences myocardial microRNA expression in atrial myocardium during coronary bypass surgery. Circulation Research, 114, 851-859. https://doi.org/10.1161/CIRCRESAHA.114.302751

Song, Y., Song, J.W., Lee, S., Jun, J.H., Kwak, Y.L., & Shim, J.K. (2017). Effects of remote ischemic preconditioning in patients with concentric myocardial hypertrophy: A randomized, controlled trial with molecular insights. International Journal of Cardiology, 249, 36-41. https://doi.org/10.1016/j.ijcard.2017.08.073

Szmagala, P., Morawski, W., Krejca, M., Gburek, T., & Bochenek, A. (1998). Evaluation of perioperative myocardial tissue damage in ischemically preconditioned human heart during aorto coronary bypass surgery. Cardiovascular Surgery, 39, 791-795.

Teoh, L.K., Grant, R., Hulf, J.A., Pugsley, W.B., & Yellon, D.M. (2002). A comparison between ischemic preconditioning, intermittent cross-clamp fibrillation and cold crystalloid cardioplegia for myocardial protection during coronary artery bypass graft surgery. Cardiovascular Surgery, 10, 251-255. https://doi.org/10.1016/S0967-2109(02)00007-8

Thielmann, M., Kottenberg, E., Kleinbongard, P., Wendt, D., Gedik, N., Pasa, S., et al. (2013). Cardioprotective and prognostic effects of remote ischaemic preconditioning in patients undergoing coronary artery bypass surgery: a single-centre randomised, double-blind, controlled trial. The Lancet, 382, 597-604. https://doi.org/10.1016/S0140-6736(13)61450-6

Tuter, D.S., Kopylov, P.Y., Syrkin, A.L., Glazachev, O.S., Komarov, R.N., Katkov, A.I., et al. (2018). Intermittent systemic hypoxic-hyperoxic training for myocardial protection in patients

undergoing coronary artery bypass surgery: first results from a single-centre, randomised controlled trial. Open Heart, 5, e000891. https://doi.org/10.1136/openhrt-2018-000891

Venugopal, V., Hausenloy, D.J., Ludman, A., Di Salvo, C., Kolvekar, S., Yap, J., et al. (2009). Remote ischaemic preconditioning reduces myocardial injury in patients undergoing cardiac surgery with cold blood cardioplegia: a randomised controlled trial. Heart, 95, 1567-1571. https://doi.org/10.1136/hrt.2008.155770

Wagner, R., Piler, P., Bedanova, H., Adamek, P., Grodecka, L., & Freiberger, T. (2010). Myocardial injury is decreased by late remote ischaemic preconditioning and aggravated by tramadol in patients undergoing cardiac surgery: a randomised controlled trial. Interactive Cardiovascular and Thoracic Surgery, 11, 758-762. https://doi.org/10.1510/icvts.2010.243600

Walsh, M., Whitlock, R., Garg, A.X., Legare, J.F., Duncan, A.E., Zimmerman, R., et al. (2015). Effects of remote ischemic preconditioning in high-risk patients undergoing cardiac surgery (Remote IMPACT): a randomized controlled trial. Canadian Medical Association Journal 188, 329-336. https://doi.org/10.1503/cmaj.150632

Wu, Q., Gui, P., Wu, J., Ding, D., Purusram, G., Dong, N., et al. (2011). Effect of limb ischemic preconditioning on myocardial injury in patients undergoing mitral valve replacement surgery. A randomized controlled trial. Circulation Journal, 75, 1885-1889. https://doi.org/10.1253/circj.CJ-10-1130

Wu, Z.K., Pehkonen, E., Laurikka, J., Kaukinen, L., Honkonen, E.L., Kaukinen, S., et al. (2001). The protective effects of preconditioning decline in aged patients undergoing coronary artery bypass grafting. The Journal of Thoracic and Cardiovascular Surgery, 122, 972-978. https://doi.org/10.1067/mtc.2001.117279

Xie, J.J., Liao, X.L., Chen, W.G., Huang, D.D., Chang, F.J., Chen, W., et al. (2012). Remote ischaemic preconditioning reduces myocardial injury in patients undergoing heart valve surgery: randomised controlled trial. Heart, 98, 384-388. https://doi.org/10.1136/heartjnl-2011-300860

Yildirim, F., Iskesen, I., Kurdal, A.T., Ozturk, T., Taneli, F., Gozukara, C., et al. (2016). Is "Attenuation of Oxidative Stress" helpful to understand the mechanism of remote ischemic preconditioning in cardiac surgery? Journal of Cardiothoracic and Vascular Anesthesia, 30, 134-140. https://doi.org/10.1053/j.jvca.2015.06.027

Young, P.J., Dalley, P., Garden, A., Horrocks, C., La, F.A., Mahon, B., et al. (2012). A pilot study investigating the effects of remote ischemic preconditioning in high-risk cardiac surgery using a randomised controlled double-blind protocol. Basic Research in Cardiology, 107, 256. https://doi.org/10.1007/s00395-012-0256-6

Zhong, H., Gao, Z., Chen, M., Zhao, J., Wang, F., Li, L., et al. (2013). Cardioprotective effect of remote ischemic postconditioning on children undergoing cardiac surgery: a randomized controlled trial. Paediatric Anaesthesia, 23, 726-733. https://doi.org/10.1111/pan.12181

Zhou, W., Zeng, D., Chen, R., Liu, J., Yang, G., Liu, P., et al. (2010). Limb ischemic preconditioning reduces heart and lung injury after an open heart operation in infants. Pediatric Cardiology, 31, 22-29. https://doi.org/10.1007/s00246-009-9536-9
