# Supplementary material for: Co‐morbidities and co‐medications as confounders of cardioprotection—Does it matter in the clinical setting?
Source: Br J Pharmacol. 2020 Jan 3;177(23):5252–69. doi: 10.1111/bph.14839 (PMC7680006; doi:10.1111/bph.14839)
Supplement: Supplementary file 4 — Figure S4: Forest plot of clinical studies on pharmacological conditioning, on cardioprotection in patients undergoing cardiovascular surgery and with biomarker release as end‐point to estimate ischaemia/reperfusion injury. The zero represents the mean value, and the gray bars represent the standard error of the mean for the placebo group. Closed squares represent significantly reduced infarct size (x ~ ± SEM), open squares represent nonsignificant changes. The star on the midline is used when no information was given to the standard error of the mean. BNP = brain natriuretic peptide; CK‐MB = creatine kinase (muscle‐brain); GIK = glucose‐insulin‐potassium; GR79236X = adenosine receptor agonist; (hs)Tn(I/T) = (high sensitive) troponin (I/T); n = no propofol anaesthesia; n.s. = not significant; PLA = placebo group; y = yes, propofol anaesthesia; ? = no information was given to the use of propofol [file BPH-177-5252-s004.pdf]

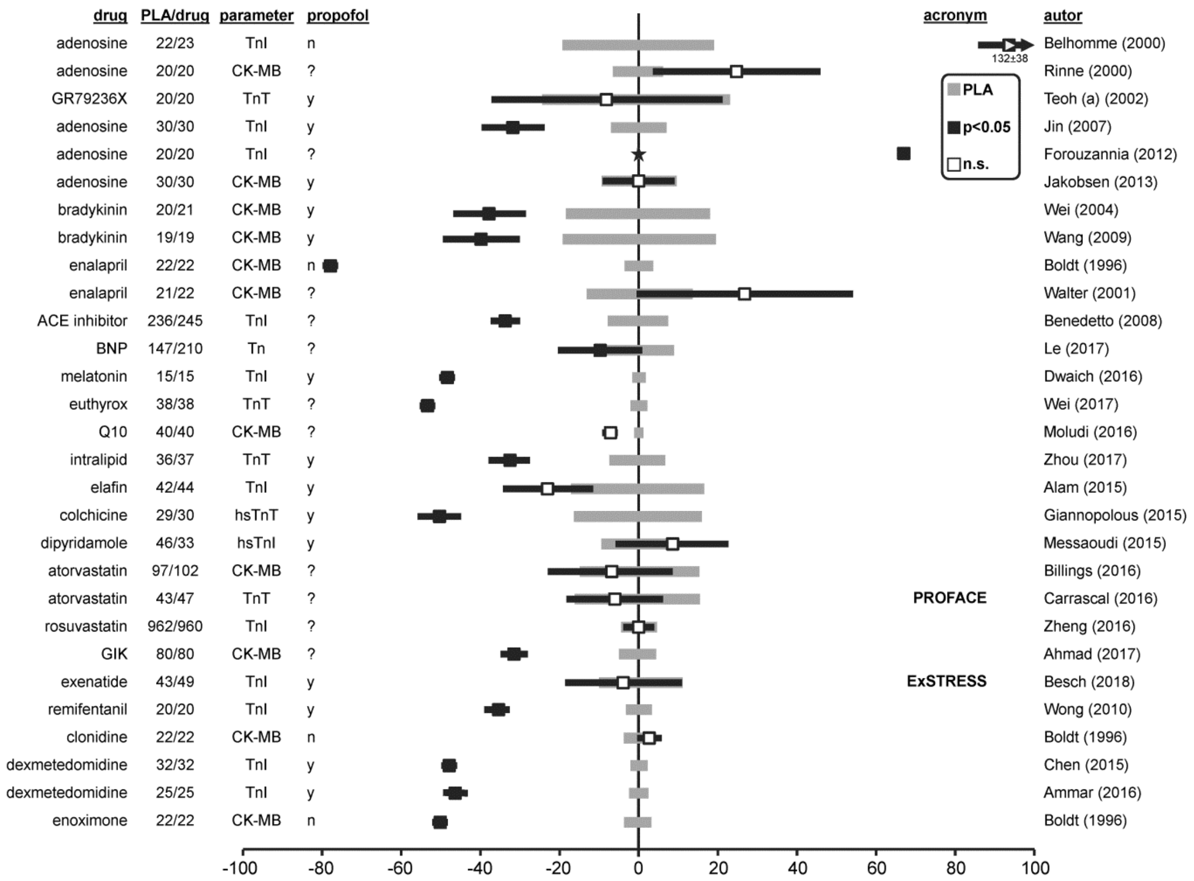


**Supplemental Figure 4:** Forest plot of clinical studies on pharmacological conditioning, on cardioprotection in patients undergoing cardiovascular surgery and with biomarker release as end-point to estimate ischaemia/reperfusion injury. The zero represents the mean value, and the gray bars represent the standard error of the mean for the placebo group. Closed squares represent significantly reduced infarct size ($\widetilde{x}$ ±SEM), open squares represent non-significant changes. The star on the midline is used when no information was given to the standard error of the mean. BNP = brain natriuretic peptide; CK-MB = creatine kinase (muscle-brain); GIK = glucose-insulin-potassium; GR79236X = adenosine receptor agonist; (hs)Tn(I/T) = (high sensitive) troponin (I/T); n = no propofol anaesthesia; n.s. = not significant; PLA = placebo group; y = yes, propofol anaesthesia; ? = no information was given to the use of propofol

## References

Ahmad, S., Ahmad, R.A., Qureshi, B.A., & Baig, M.A.R. (2017). Myocardial protection with Glucose-Insulin-Potassium infusion during adult cardiac surgery. Pakistan Journal of Medical Sciences, 33, 325-329. https://doi.org/10.12669/pjms.332.12414

Alam, S.R., Lewis, S.C., Zamvar, V., Pessotto, R., Dweck, M.R., Krishan, A., et al. (2015). Perioperative elafin for ischaemia-reperfusion injury during coronary artery bypass graft surgery: a randomised-controlled trial. Heart, 101, 1639-1645. https://doi.org/10.1136/heartjnl-2015-307745

Ammar, A.S., Mahmoud, K.M., Kasemy, Z.A., & Helwa, M.A. (2016). Cardiac and renal protective effects of dexmedetomidine in cardiac surgeries: A randomized controlled trial. Saudi Journal of Anaesthesia, 10, 395-401. https://doi.org/10.4103/1658-354X.177340

Belhomme, D., Peynet, J., Florens, E., Tibourtine, O., Kitakaze, M., & Menasche, P. (2000). Is adenosine preconditioning truly cardioprotective in coronary artery bypass surgery ? The Annals of Thoracic Surgery, 70, 590-594. https://doi.org/10.1016/S0003-4975(00)01502-2

Benedetto, U., Melina, G., Capuano, F., Comito, C., Bianchini, R., Simon, C., et al. (2008). Preoperative angiotensin-converting enzyme inhibitors protect myocardium from ischemia during coronary artery bypass graft surgery. Journal of Cardiovascular Medicine (Hagerstown), 9, 1098-1103. https://doi.org/10.2459/JCM.0b013e32830a6daf

Besch, G., Perrotti, A., Salomon du Mont, L., Puyraveau, M., Ben-Said, X., Baltres, M., et al. (2018). Impact of intravenous exenatide infusion for perioperative blood glucose control on myocardial ischemia-reperfusion injuries after coronary artery bypass graft surgery: sub study of the phase II/III ExSTRESS randomized trial. Cardiovascular Diabetology, 17, 140. https://doi.org/10.1186/s12933-018-0784-y

Billings, F.T. 4th, Hendricks, P.A., Schildcrout, J.S., Shi, Y., Petracek, M.R., Byrne, J.G., et al. (2016). High-dose perioperative atorvastatin and acute kidney injury following cardiac surgery: A randomized clinical trial. Journal of the American Medical Association, 315, 877-888. https://doi.org/10.1001/jama.2016.0548

Boldt, J., Rothe, G., Schindler, E., Döll, C., Görlach, G., & Hempelmann, G. (1996). Can clonidine, enoximone, and enalaprilat help to protect the myocardium against ischaemia in cardiac surgery? Heart, 76, 207-213.

Carrascal, Y., Arnold, R.J., De la Fuente, L., Revilla, A., Sevilla, T., Arce, N., et al. (2016). Efficacy of atorvastatin in prevention of atrial fibrillation after heart valve surgery in the PROFACE trial (PROphylaxis of postoperative atrial Fibrillation After Cardiac surgEry). Journal of Arrhythmia, 32, 191-197. https://doi.org/10.1016/j.joa.2016.01.010

Chen, S., Hua, F., Lu, J., Jiang, Y., Tang, Y., Tao, L., et al. (2015). Effect of dexmedetomidine on myocardial ischemia-reperfusion injury. International Journal of Clinical and Experimental Medicine,

8, 21166-21172.

Dwaich, K.H., Al-Amran, F.G., Al-Sheibani, B.I., & Al-Aubaidy, H.A. (2016). Melatonin effects on myocardial ischemia-reperfusion injury: Impact on the outcome in patients undergoing coronary artery bypass grafting surgery. International Journal of Cardiology, 221, 977-986. https://doi.org/10.1016/j.ijcard.2016.07.108

El Messaoudi, S., Wouters, C.W., van Swieten, H.A., Pickkers, P., Noyez, L., Kievit, P.C., et al. (2016). Effect of dipyridamole on myocardial reperfusion injury: A double-blind randomized controlled trial in patients undergoing elective coronary artery bypass surgery. Clinical Pharmacology and Therapeutics, 99, 381-389. https://doi.org/10.1002/cpt.106

Forouzannia, S.K., Abdollahi, M.H., Mirhosseini, S.J., Hadadzadeh, M., Zarepur, R., Zarepur, E., et al. (2013). Adenosine preconditioning versus ischemic preconditioning in patients undergoing off-pump coronary artery bypass (OPCAB). The Journal of Tehran Heart Center, 8, 127-131.

Giannopoulos, G., Angelidis, C., Kouritas, V.K., Dedeilias, P., Filippatos, G., Cleman, M.W., et al. (2015). Usefulness of colchicine to reduce perioperative myocardial damage in patients who underwent on-pump coronary artery bypass grafting. The American Journal of Cardiology, 115, 1376-1381. https://doi.org/10.1016/j.amjcard.2015.02.036

Jakobsen, O., Naesheim, T., Aas, K.N., Sorlie, D., & Steensrud, T. (2013). Adenosine instead of supranormal potassium in cardioplegia: it is safe, efficient, and reduces the incidence of postoperative atrial fibrillation. A randomized clinical trial. The Journal of Thoracic and Cardiovascular Surgery, 145, 812-818. https://doi.org/10.1016/j.jtcvs.2012.07.058

Jin, Z.X., Zhou, J.J., Xin, M., Peng, D.R., Wang, X.M., Bi, S.H., et al. (2007). Postconditioning the human heart with adenosine in heart valve replacement surgery. Annals of Thoracic Surgery 83, 2066-2072. https://doi.org/10.1016/j.athoracsur.2006.12.031

Le, S., Xiao, J., Li, W., Wang, J., Wang, Q., Xi, W., et al. (2017). Continuous administration of recombinant human B-type natriuretic peptide can improve heart and renal function in patients after cardiopulmonary bypass surgery. Journal of Thoracic Disease, 9, 692-701. https://doi.org/10.21037/jtd.2017.03.20

Moludi, J., Keshavarz, S., Tabaee, A.S., Safiri, S., & Pakzad, R. (2016). Q10 supplementation effects on cardiac enzyme CK-MB and troponin in patients undergoing coronary artery bypass graft: a randomized, double-blinded, placebo-controlled clinical trial. Journal of Cardiovascular and Thoracic Research, 8, 1-7. https://doi.org/10.15171/jcvtr.2016.01

Rinne, T., Laurikka, J., Penttila, I., & Kaukinen, S. (2000). Adenosine with cold blood cardioplegia during coronary revascularization. Journal of Cardiothoracic and Vascular Anesthesia, 14, 18-20. https://doi.org/10.1016/S1053-0770(00)90049-1

Teoh, L.K.K., Grant, R., Hulf, J.A., Pugsley, W.B., & Yellon, D.M. (2002). The effect of preconditioning (ischemic and pharmacological) on myocardial necrosis following coronary artery bypass graft surgery. Cardiovascular Research, 53, 175-180. https://doi.org/10.1016/S0008-6363(01)00435-7

Walter, D.H., Fichtlscherer, S., Britten, M.B., Rosin, P., Auch-Schwelk, W., Schächinger, V., et al. (2001). Statin therapy, inflammation and recurrent coronary events in patients following coronary stent implantation. Journal of the American College of Cardiology, 38, 2006-2012.

Wang, X., Wei, M., Kuukasjarvi, P., Laurikka, J., Rinne, T., Moilanen, E., et al. (2009). The anti-inflammatory effect of bradykinin preconditioning in coronary artery bypass grafting (bradykinin and preconditioning). Scandinavian Cardiovascular Journal, 43, 72-79. https://doi.org/10.1080/14017430802180449

Wei, M., Wang, X., Kuukasjarvi, P., Laurikka, J., Rinne, T., Honkonen, E.L., et al. (2004). Bradykinin preconditioning in coronary artery bypass grafting. Annals of Thoracic Surgery 78, 492-497. https://doi.org/10.1016/j.athoracsur.2003.11.039

Wei, Q.B., Xie, F., Wang, S.L., & Li, G. (2017). Effect of thyroid hormone on myocardial and cerebral ischemia reperfusion injury in valve replacement under cardiopulmonary bypass. Journal of Hainan Medical University, 23, 79-82.

Wong, G.T., Huang, Z., Ji, S., & Irwin, M.G. (2010). Remifentanil reduces the release of biochemical markers of myocardial damage after coronary artery bypass surgery: a randomized trial. Journal of Cardiothoracic and Vascular Anesthesia, 24, 790-796. https://doi.org/10.1053/j.jvca.2009.09.012

Zheng, Z., Jayaram, R., Jiang, L., Emberson, J., Zhao, Y., Li, Q., et al. (2016). Perioperative rosuvastatin in cardiac surgery. The New England Journal of Medicine, 374, 1744-1753. https://doi.org/10.1056/NEJMoa1507750

Zhou, R.H., Yu, H., Yin, X.R., Li, Q., Yu, H., Yu, H., et al. (2017). Effect of intralipid postconditioning on myocardial injury in patients undergoing valve replacement surgery: a randomised controlled trial. Heart, 103, 1122-1127. https://doi.org/10.1136/heartjnl-2016-310758
